# A pragmatic adaptive trial of hope-focused mentoring to improve mental health and social outcomes for young women who are not in education, employment or training in deprived coastal areas (The Looking Forward Project): feasibility trial stage protocol

**DOI:** 10.1186/s40814-026-01852-4

**Published:** 2026-05-30

**Authors:** Clio Berry, Jessica C Bird, Stephen Bremner, Saskia Eddy, Lindsay Forbes, Julia Fountain, Paul McCrone, Charlotte Rawlinson, Jon Wilson, Daniel Michelson

**Affiliations:** 1 Department of Primary Care and Public Health, Brighton and Sussex Medical School, University of Brighton and University of Sussex, Brighton, BN1 9PX UK; 2 Department of Child & Adolescent Psychiatry, Institute of Psychiatry, King’s College London, Psychology & Neuroscience, Strand, London, UK; 3 Division of Methodologies, Faculty of Nursing, Midwifery & Palliative Care, King’s College London, London, UK; 4 Centre for Health Services Studies, University of Kent, Canterbury, Kent UK; 5 Research and Development, Sussex Education Centre, Sussex Partnership NHS Foundation Trust, Nevill Avenue, Hove, UK; 6 School of Health Sciences, University of Greenwich, London, UK; 7 Norfolk and Suffolk NHS Foundation Trust, Norwich, UK; 8 NIHR Maudsley Biomedical Research Centre, South London and Maudsley NHS Foundation Trust and King’s College London, London, UK

**Keywords:** Pilot, Women’s health, Young people, Positive youth development, Mental health

## Abstract

**Background:**

A large number of young women are not in education, employment, or training (NEET) in England. This group experiences poorer mental health and social outcomes than young women who work and study, and NEET young men. Gender disparities are compounded in deprived and coastal areas, where young women especially lack self-agency and aspirations. Research suggests that greater hope lowers risks of staying NEET and developing mental health problems. We co-developed a hope-focused intervention (HOPEFUL) for this group. HOPEFUL is a flexible modular programme, designed to help develop a more hopeful mindset that facilitates setting and pursuing personally meaningful goals. It is designed to be supported by a mentor, if possible selected by the participant from their existing social network. We aim to understand the feasibility of involving NEET young women and mentors in a trial of HOPEFUL conducted in coastal and adjacent local authority areas in Sussex, Kent and Medway, and East Anglia.

**Methods:**

This protocol describes the feasibility stage of an adaptive assessor-blind pragmatic randomised controlled trial (RCT) with 1:1 allocation (stratified by age group and local authority area) to (1) mentor-supported HOPEFUL plus usual support versus (2) usual support with waitlist access to HOPEFUL materials. NEET young women (aged 16–25 years) are recruited from local authority, charity and voluntary, and primary care services, as well as directly from the community using outreach, local advertising, social media, and peer networks. Our adaptive trial design incorporates a preliminary feasibility trial (*N* = 70) with progression to a definitive RCT (*N* = 248), as warranted. Feasibility outcomes (corresponding to the recruitment and retention of young women and mentors) will be assessed post-intervention (16 weeks) against pre-specified progression criteria.

**Discussion:**

Feasibility trial results will inform decisions about progression to a definitive RCT of the HOPEFUL intervention. If warranted, the latter will test whether HOPEFUL improves hope (primary outcome) and secondary outcomes (including mental health symptoms and social outcomes) compared to usual support services at 16 weeks (primary endpoint) and 12-month follow-up. We will disseminate feasibility results using tailored outputs. In the longer term, we anticipate practice impacts through scaled-up access to HOPEFUL.

**Supplementary Information:**

The online version contains supplementary material available at 10.1186/s40814-026-01852-4.

## Background

The number of young women (16–25 years) who are not in education, employment, or training (NEET) in England is high and significantly growing, and this group experiences worse long-term mental health and social outcomes relative to young women who work and/or study [1–3]. Compared to NEET young men, NEET young women also have poorer mental and physical health [1, 3], more chronic health conditions [1], greater suicidality and self-harm [4], greater social isolation [5, 6], and are more likely to stay NEET due to mental ill-health [7]. Time spent NEET can have enduring “scarring” effects, predicting mental health problems and unemployment decades later [8]. The multiple disadvantages for NEET young women are compounded in deprived areas [9], such as England’s coastal areas that have fewer jobs, more underperforming schools, poorer infrastructure, and lower aspirations than even deprived inland regions [10]. Whilst influential, these structural factors do not dictate life trajectories [11] and varying outcomes of NEET subgroups are mediated by differences in self-beliefs and appraisals [12, 13].

Developmental work leading to the current trial focused on “hope” as a key interventional lens for NEET young women living in coastal deprivation [14]. Hope can be defined as the belief that one can reach personally meaningful goals (self-agency) and identify how to do so (pathways) [15]. Greater hope predicts improved social and mental health [16–19], including reduced depression [16], greater resilience [20], reduced impacts of adversity [11, 21], and reduced suicidality [22]. Hope also increases help-seeking [16] and subsequent engagement with care for mental health problems and suicidality [23]. In addition, hope predicts positive academic and employment outcomes [16–18], more strongly than past performance, IQ, cognitive ability, personality, and self-efficacy [24, 25]. Crucially, evidence additionally shows that people with greater hope are less likely to become NEET or stay NEET over time [11].

NEET young people have less hope than other groups on average [11] and this is especially true for NEET young women [26]. Young women moreover particularly lack hope relative to young men when they live in deprived and geographically remote places [27], such as coastal areas. NEET young women’s hope is further undermined by interpersonal discontinuity in youth services [14] and pessimistic messaging from their families and communities about possible futures [11, 14]. Hope is a psychologically precise, self-reinforcing construct [16, 28], with broad impacts on how people engage with their environments, making it a promising intervention target [29]; particularly for NEET young women in deprived coastal communities where enhancing hope may be especially beneficial.

A systematic review by our group showed that even brief interventions delivered by non-specialists in non-clinical settings can enhance vulnerable young people’s hope and provide additional mental health and social benefits [16]. We additionally found that hope can be reliably measured to show such effects [16]. Building on this review, we co-designed the HOPEFUL intervention: a hope-focused programme with mentoring support for NEET young women living in coastal areas. Our co-design process involved working with NEET young women, their relatives, and professionals from a variety of health, youth, and education support organisations [14].

HOPEFUL is a flexible modular programme that uses psychoeducational components, lived experience stories, and a menu of interactive activities to help young women to increase their hope. Co-design participants endorsed the potential of an innovative “youth-initiated mentoring” model [30, 31] in which a prospective mentee nominates a mentor, such as a non-parental relative or sports coach, from within their existing network. International evidence suggests that youth-initiated mentoring improves mental health and social functioning for disadvantaged youth [30, 31]. Although seemingly untested in the UK, this practice model is highly relevant to England’s under-resourced coastal areas, for it requires fewer resources than professional mentoring (or other professional-led interventions) [30, 31], whilst having the potential to build community capacity for supporting vulnerable young people in the longer term.

### Aims and research questions

Our overarching aim is to evaluate the acceptability, effectiveness, and cost-effectiveness of HOPEFUL delivered to NEET young women in deprived coastal areas. We plan to conduct a randomised controlled trial (RCT) that evaluates hope (primary outcome) and mental health and social outcomes, and must first ensure that this is feasible. In this feasibility trial stage, we aim to address the following research questions:

1. Is it feasible to recruit and retain, with complete data, NEET young women and mentors in an RCT of HOPEFUL plus usual support versus usual support alone?
2. Can HOPEFUL be delivered as intended?
3. Do NEET young women and mentors form positive mentoring relationships?
4. How do NEET young women and mentors experience participation in the HOPEFUL intervention and associated research procedures?
5. What is the estimated standard deviation (SD) of the Trait Hope Scale (primary outcome) in NEET young women?
6. What do NEET young women consider to be the minimum meaningful change in this measure of hope?
7. What changes, if any, are needed to increase acceptability and feasibility of the intervention and research protocols?

Data will be collected on feasibility parameters to determine progression to the RCT, using the pre-specified progression criteria described below. Qualitative interviews with participants will provide more detailed insights into feasibility and acceptability.

## Methods

### Trial design

The trial was registered in the ISRCTN registry on the 10th September 2024, reference ISRCTN52288029. Recruitment began in October 2024 and was ongoing at the time of submitting this manuscript.

Our evaluation approach draws on Medical Research Council (MRC) guidance for evaluating complex interventions [32, 33], testing feasibility before evaluating whether our intervention works, how, and for whom. Following from Snyder’s cognitive model [28], we conceptualise hope as goal-directed, comprising belief in one’s capacity to work toward their goals and the ability to identify pathways for reaching them. Our previous work [14, 16, 34] suggests that this model aligns with young people’s “lay” conceptualisations of hope. We additionally situate HOPEFUL within the “wise” intervention approach [29, 35], which focuses on targeting specific psychological processes to improve health through influencing how people interact with their environments [29]. The practical specification of the HOPEFUL intervention was shaped through extensive co-design activities with intended “end users”, in line with the person-based approach to intervention development [14, 36].

The evaluation design is an adaptive, assessor-blind, pragmatic, controlled superiority, parallel arm, randomised controlled trial. It incorporates two stages: the feasibility stage (described in this protocol) followed by a definitive RCT stage, if warranted. Randomisation will use a 1:1 allocation ratio stratified by relevant local authority area and age (16 to 18, 19 to 25 years). The two arms are (1) HOPEFUL assisted by a mentor plus usual support (the “HOPEFUL Together” intervention arm), which will be compared with (2) delayed access to the HOPEFUL workbook plus usual support (the “HOPEFUL Future” control arm).

Effectiveness outcomes in the putative definitive trial will be collected at baseline, post-intervention (16 weeks; primary endpoint), and follow-up (12 months; secondary endpoint). Feasibility analysis will use data from two outcome assessment points, baseline and 16 weeks. The adaptive component of this trial is that the feasibility participants’ outcome data will be subsumed into the definitive trial sample, unless feasibility results indicate that this should not occur. Feasibility data will be used alongside pre-specified progression criteria to determine the appropriate continuation scenario.

### Study setting

As per 2016 Department for Education guidance [37], English local authorities have statutory duties to monitor and increase young people’s participation in education, employment, or training. The focus of commissioning for NEET young people is children’s services, where case management is generally provided up to age 18 years. This increases to age 25 years for people with special educational needs, mental health problems, or care experience. Some local authorities, voluntary services, and charities provide additional support for young people up to 25 years, including counselling, mentoring, and advocacy. Relevant third-sector services may be fully or partly commissioned by local authorities.

The trial will run in Sussex, Kent and Medway, and East Anglia, focusing on local authority areas containing a coastline or estuary as well as adjacent inland local authority areas within the same geographical county. This pragmatic approach reflects the absence of a consensus definition for the term “coastal” in public health, civic, or demographic contexts [38]. Authorities in Sussex, Kent and Medway, and East Anglia report above average and rising prevalence of NEET populations over the past 5 years [2]. We will concentrate recruitment on neighbourhoods within the local authority areas that are deprived, but any eligible NEET young women within the involved local authority areas will be able to participate.

### Eligibility criteria

Eligibility will be based on the following:

1. Aged 16 to 25 years at time of consent,
2. Identify as a woman,
3. NEET, operationalised as having no involvement in education, employment, or training (EET) activity in the past month as measured using the Time Use Survey [39],
4. Residing in a local authority area involved in the study, and
5. Able to give informed consent.

The third criterion (NEET status) will not preclude informal activities such as casual babysitting, or one-off activities such as waiting tables at a single event. Potential participants will be excluded based on elevated suicidality, operationalised as a score of non-zero on the suicidality item of the Patient Health Questionnaire (PHQ-9) [40] plus a rating of four or more out of seven with respect to severity of the suicidality, as measured by a screening version of the Columbia-Suicide Severity Rating Scale (C-SSRS) [41].

Eligible mentors will be (1) aged 18 years or more at time of consent, and (2) able to give informed consent. Each mentor will be either identified by the young woman or alternatively allocated by the study team, as described further in the “Interventions” and “Recruitment” sections below. There will be no exclusion criteria for mentors. However, young women will be supported to nominate appropriate mentors: a non-parent and someone with whom they have a safe and non-conflictual relationship (see the “Interventions” section for further details).

### Interventions

#### Intervention arm (HOPEFUL Together)

This arm comprises the use of the HOPEFUL programme with support from a mentor. Participants will also have access to usual support for NEET young women (see description in comparator arm below). HOPEFUL is a six-module psychosocial intervention programme including psychoeducational, cognitive-behavioural, and interpersonal components. The intervention Theory of Change is provided in Table 1, and reported using TiDIER [42] categories below.

**Table 1:** HOPEFUL intervention Theory of Change

HOPEFUL Theory of Change model								
**Problem:** Young women who are not in education, employment, and training (NEET) lack hope and are at risk of potentially long-term mental health problems and social exclusion. **Focus:** Enhancing hope through a structured psychosocial intervention, delivered using online and printed materials with support from a mentor.								
**Inputs**	**Outputs**			**Outcomes**				
	**Participation**	**Activities**	**Change in outcome category facilitated by (module)**	**Mechanisms of outcome**	**Category**	**Short term (0 to 6 months)**	**Medium term (> 6 to 12 months)**	**Long term (> 12 months to 5 years)**
**People and services** • Mentors • Mentor trainers/supervisors • Referrals initiated by relevant statutory and community and voluntary sector organisations and/or directly from NEET young women **Resources and materials** • Workbook (online/paper) for young woman, presenting six modules; each focusing on a specific topic related to hope and containing a menu of activities • Training resources, supervision, and manual for mentors	• Young women who are NEET and living in deprived and coastal communities • Youth-initiated mentor for each young woman (or alternative mentor identified if needed)	**HOPEFUL intervention delivery** • 1:1 psychosocial support, guided by a mentor, according to a structured but flexible manualised programme • Active encouragement to spend time outside of the home (e.g., walking conversations; behavioural activation) • Completion of workbook by young person • Group component possible, e.g., toward end of the intervention • Basic needs identified and addressed **Training and supervision** • Provided to mentors using scalable formats (e.g., video-based training; group supervision) that and proportionate to intervention requirements	• Hopeful mentoring relationship (1–6) • Behavioural activation (1–6) • Learning about hope and its sources (2) • Practising skills in goal setting and pursuit (4) • Increasing positive social relationships (5)	• Personal goal attainment, Goal-Based Outcome Tool • Quality of mentoring relationship, Working Alliance Inventory	**Hope**	• Improved motivation^a^ • Recognise the importance of setting achievable goals^a^ • Improved confidence to change behaviour^a^ • Ability to set realistic goals and develop plans^a^	• Raised life aspirations	• Sustained increase in hope^a^ • Sustained raised life aspirations
			• Boosting time spent in activities outside the home (1–6) • Identifying interests and strengths (1) • Change in mental health and well-being is additionally facilitated by the outcome of increased hope		**Mental health and well-being**	• Increased self-awareness and ability to reflect^b^ • Improved sense of identity	• Reduced anxiety^c^ • Reduced depression^d^ • Reduced suicidality^d^ • Decreased mental health support needs^c,d,e^	• Improved resilience • Reduced alcohol and substance misuse • Reduced contact with youth custody/criminal justice orders^e^ • Decreased mental health service use, including A&E^e^
			• Hopeful mentoring relationship (1–6) • Identifying interests, strengths and values (1, 3) • Practising skills in goal setting and pursuit (4) • Increasing social capital via social relationships (5) • Change in EET is additionally facilitated by increased hope		**Employment, education and training (EET)**	• Improved capability (skills and interests)	• Increased awareness of careers and access routes • Re/entered EET^f^	• Maintaining meaningful EET^f^ • Motivation to upskill, seek promotions and continue to develop
			• Positive and consistent mentoring relationship, and any group activities (1–6) • Practising communication skills (5) • Understanding characteristics of hopeful relationships (5) • Change in social functioning additionally facilitated by increased hopefulness		**Social functioning**	• Improved social skills • Increased opportunities to form meaningful friendships^g^ • Increased levels of trust in social relationships • Expanded social networks and support systems^g^	• Increased network of support and positive relationships with other people^g^	• Improvement in parenting skills
			• Supportive relational environment through mentor (1–6) • Increasing positive social relationships (5) • Practising communication skills (5) • Change in help-seeking outcomes additionally facilitated by increased hope		**Help-seeking**	• Improved knowledge of available support and how to access it^h^	• Confidence to seek and ask for the right type of support^h^	• Sustained confidence to seek and ask for the right type of support^h^

Assumptions: Capacity in existing services to train/supervise mentors; HOPEFUL intervention is viewed by stakeholders as an acceptable and feasible intervention

As measured by: ^a^Trait Hope Scale; ^b^Short Warwick-Edinburgh Mental Well-Being Scale; ^c^Patient Health Questionnaire; ^d^Generalised Anxiety Disorder Scale;^e^Client Service Receipt Inventory; ^f^Time Use Survey; ^g^UCLA Loneliness Scale; ^h^General Help-Seeking Questionnaire

##### Why

The explicit primary focus of HOPEFUL is hope, drawing primarily on cognitive hope theory [28]. During our developmental work [14], NEET young woman perceived hope as a more novel, engaging, sensitive, and relevant primary intervention target than either mental health or EET outcomes. Nevertheless, associated improvements are expected in mental health and socio-occupational outcomes (including engagement in EET) and these will be measured as secondary outcomes.

##### What

HOPEFUL will be offered as a paper workbook and a digital version, hosted on the behopeful.co.uk website and accessible via log-in details that will be provided separately to NEET participants and their mentors. HOPEFUL is organised into six modules, each containing core psychoeducational material and a menu of activities to help with applying newly learned concepts and skills in practice. Use of the workbook will be supported by a mentor (see the “Who provides” section). Mentors will also able to access two training videos (approximately 60 min in total) on the HOPEFUL website.

##### Where

The intervention will be accessed through multiple services and routes, including referrals from health, education, and other support services, and self-referrals following promotion in diverse community venues (e.g., foodbanks) and online. Mentoring sessions can take place in any location agreed upon by the participant and their mentor. Participants and mentors will be encouraged, where appropriate, to meet in suitable outdoor locations, incorporating physical activity and nature exposure (e.g., walking conversations with mentors). Mentoring sessions may also be conducted online or by telephone, depending on participant convenience and preference.

##### When and how much

The intervention is designed to be delivered flexibly. Young women may meet their mentor in regular sessions and/or use the workbook in a self-directed fashion. The precise number and spacing of mentoring sessions will be agreed collaboratively between the mentor and participant. As a guide, mentoring should occur typically over 4 to 12 sessions (meetings), each lasting approximately 30 to 90 min, spaced about 3 to 14 days apart, over an approximate period of 4 to 12 weeks.

##### Tailoring

Before engaging with the six intervention modules, there is an introductory component in which the young woman and mentor agree a set of guidelines for their mentoring relationship. The six modules can be used in sequence, although it is also possible to skip/re-order modules if preferred. Module activities can be completed flexibly using role play, discussions, creative arts, writing, outdoor activities, and/or self-study. The first module (About Me) focuses on building positive sense of self and increasing time spent in meaningful activity. Module 2 (About Hope) focuses on exploring young women’s own sense of hope and where it comes from. Modules 3 to 6 (My Values, My Goals, My Hope Network, Staying Hopeful) focus on learning and practising skills for identifying, setting, and pursuing goals, and overcoming barriers. Each module has a core psychoeducational component and a relevant lived experience story from a young person, both presented in animated video format. Each module includes a menu of suggested session activities to practise key skills, along with optional activities for young women to complete independently. Each module also includes a “share sheet” to help young women explain the focus of the module to others and ask for support in continuing to use the skills they have learned.

##### Who provides

Use of the HOPEFUL programme is designed to be supported by a mentor, with efforts made at the outset to support the young women to identify a youth-initiated mentor, i.e., someone the young woman already knows and trusts (at least one “back-up”) as part of their baseline assessment session. A researcher will show an animated video that describes mentoring and guides the young women as to selecting an appropriate mentor, who they consider to be hopeful, supportive, respectful, and reliable. It cautions against selecting a parent or anyone with whom the relationship feels confusing, critical, unsafe, or abusive. The video also suggests selecting someone who is able to meet regularly and in person. Following the video, a researcher will use a structured proforma to invite nominations for potential mentors and to check they are appropriate. If a young woman is unable to nominate a mentor from their existing social network, or if their nominated mentor declines, an alternative will be allocated by the study team. These study-allocated mentors will be selected from a pool of professionals and community volunteers on a case-by-case basis, considering the young woman’s locality. Young women may also forego a mentor entirely and use the workbook independently if that is their preference.

The mentor’s role—which is the same regardless of whether youth-initiated or study-allocated—is to provide supportive accountability [43], i.e., encouraging the young woman to use HOPEFUL and to aid their understanding of the components as needed. The guiding principles of the mentor’s approach are that it is hopeful, open, patient, engaging, flexible, understanding, and light-hearted. Specialist knowledge or technical skills are not expected or required. The manual and training introduce the role and the guiding principles, and provide role-plays, “troubleshooting” guidance, and further information resources. Before starting mentoring, prospective mentors meet with a senior member of the research team to ensure they have completed their training and to review its content, address any questions, and support them to begin the mentoring process.

All mentors will be paired with a supervisor, drawn from a pool of local authority and voluntary sector staff (e.g., experienced youth and employability workers) in the project regions. Mentor supervisors will receive a manual and training (approximately 90 min) to help them understand the mentor’s role and likely challenges. Supervisors have an on-boarding call with the research team, to ensure training has been completed and review its content, and have ongoing consultation with the research team. Supervisors are encouraged to provide supervision to mentors approximately fortnightly. In the event of delays or issues in accessing supervision, it will be provided by the research team.

#### Control arm (HOPEFUL Future)

This arm comprises usual support plus waitlist access to the HOPEFUL workbook for self-directed use. Usual support varies from nothing to support from social services, educational or employment services, and/or primary care and/or specialist mental health services. We will aim to standardise this support by providing young women with information about local relevant provision. Participants will also have the option to approach their nominated mentor(s) for informal support, although no supervision or other external assistance will be provided for this mentoring relationship. Data on usual support provision will be collected using an adapted Client Service Receipt Inventory (CSRI [44]) at baseline (covering the previous 6 months) and at follow-up (covering the time since the last assessment point) [44].

A waitlist control design was chosen since NEET young women often experience barriers to accessing support and may feel neglected by services [14]. At the end of their participation in the trial, we will offer access to the HOPEFUL workbook, which young women may choose to use with their nominated mentor(s), again without supervision or additional structured support.

### Outcomes

#### Quantitative data

Data will be collected on the following feasibility parameters and assessed against pre-specified progression criteria (see Table 2).

- Numbers and proportions of NEET young women identified, interested, consented, eligible, and randomised by area per month.
- Number and proportion of scheduled HOPEFUL mentoring sessions completed by NEET young women (HOPEFUL Together arm only).
- Number and proportion of participants retained in the trial and completing 16-week assessments (young women in both arms).
- Data completeness for baseline and 16-week assessments (young women in both arms).

**Table 2:** Pre-specified progression criteria

Green	Amber	Red
Recruitment (i.e., consent) of 100% (*n* = 70) of the target of NEET young women within the specified recruitment period	Recruitment of 50 to less than 100% (*n* = 35–69) of the target of NEET young women within the specified recruitment period	Recruitment of less than 50% (*n* ≤ 34) of the target of NEET young women within the specified recruitment period
At least 40% of young people identified during the recruitment process are eligible and interested in participation	30 to less than 40% of young people identified during the recruitment process are eligible and interested in participation	Less than 30% of young people identified during the recruitment process are eligible and interested in participation
At least 60% of NEET young women allocated to HOPEFUL complete 4 or more sessions	40 to less than 60% of young women allocated to HOPEFUL complete 4 or more sessions	Less than 40% of NEET young women allocated to HOPEFUL complete 4 or more sessions
At least 80% of participants provide primary outcome data at post-intervention assessment	50 to less than 80% of participants provide primary outcome data at post-intervention assessment	Less than 50% of participants provide primary outcome data at post-intervention assessment

We will additionally record the numbers and proportions of youth-initiated mentors identified (and consented and trained), and the numbers and proportions of supervision sessions attended by mentors, though these data will not be directly considered as progression criteria.

Participants in our developmental study strongly advocated hope as the primary outcome to assess effectiveness [14]. The Trait Hope Scale (THS) [15] was identified in our previous systematic review [16] with evidence that it can increase following brief interventions across diverse populations and contexts [16]. Relevant secondary outcomes were also identified by young women and professionals and specified in our co-produced Theory of Change (see Table 1) [14]. To assess mental health outcomes, we selected the PHQ-9 and GAD-7, which are used routinely in the UK’s Improving Access to Psychological Therapies (IAPT), services, and are recommended internationally by the Wellcome Trust [45] as common metrics in psychological intervention trials. These and other outcome measures for the definitive RCT are listed below and will also be collected in the feasibility stage.

1. Primary outcome: Hope measured with the 12-item self-report Trait Hope Scale (THS) [15];
2. Secondary outcomes:
  Well-being will be measured using the 7-item Short Warwick-Edinburgh Mental Well-Being Scale (SWEMWBS) [46, 47];
  Depressive symptoms will be measured using the 9-item self-report Patient Health Questionnaire (PHQ-9) [40];
  Anxiety symptoms will be measured using the 9-item self-report Generalised Anxiety Disorder scale (GAD-7) [48];
  Social anxiety will be measured using a 12-item self-report [49] scale that combines Social Interaction Anxiety Scale short form (SIAS-6) and Social Phobia Scale short form (SPS-6);
  Meaning in life will be measured using the 10-item self-report Meaning in Life Questionnaire (MLQ) [50];
  Time spent in structured activity will be measured using time spent in Education, Employment, and Training (EET), plus other constructive economic (childcare, housework, and chores) and structured (sports and structure leisure) activities, as captured with an adapted Time Use Survey (TUS) [39, 51];
  Loneliness will be measured using the short 8-item self-report UCLA Loneliness Scale (UCLA-8) [52];
  Global socio-occupational functioning will be measured using the assessor-rated Social and Occupational Functioning Scale (SOFAS) [53];
  Help-seeking will be measured using the 10-item self-report General Help-Seeking Questionnaire (GHSQ) [54];
  Adverse effects for participants in HOPEFUL Together arm will be measured using the self-report modified Edinburgh Adverse Effects of Psychological Therapy Scale (EDAPTS) [55];
  Adverse events (AEs) will be measured using a study-specific Adverse Events Checklist, completed by the research team;
3. Economic outcomes will be measured using the brief semi-structured Client Service Receipt Inventory (CSRI) [44] questionnaire, adapted to capture statutory and broader support, and by calculating mental well-being adjusted life years using the SWEMWBS [46] value set;
4. Proposed mechanisms of intervention effects in the HOPEFUL Together arm will be measured using attainment for three personally identified goals: self-rated 3-item idiographic Goal-Based Outcome Tool (GBOT) [56] collected during intervention modules four to six, and a short revised 12-item Working Alliance Inventory (WAI-SR) [57] completed by NEET young women and mentors (separately) approximately after intervention session three; and
5. Mentor outcomes in the HOPEFUL Together arm will be measured using the THS [15] and SWEMWBS [47] at baseline and follow-up.

We will collect data separately on adverse effects (i.e., undesired and potentially harmful effects of an intervention) and adverse events (i.e., untoward changes or events that are not specific to intervention). Further details are provided in the “Adverse events” section below. In addition, two brief neurocognitive assessments will be conducted at baseline to describe and contextualise the trial sample with respect to neurocognitive functioning, as compared to population norms. These assessments comprise tests of verbal fluency (Controlled Oral Word Association Test (COWAT) [58] for the letters F, A, and S) and verbal memory (Morris Revision IV [59] of the Logical Memory Scale).

#### Qualitative data

Qualitative data will be collected within individual interviews with young women and mentors after their 16-week follow-up assessments. Trained peer researchers, who are young women with experience of being NEET, and unblinded trial staff will collect qualitative data from young women and mentors respectively. Data will be collected within semi-structured interviews, using topic guides reviewed by our Public Involvement Panel (PIP). The interviews will explore acceptability parameters [60], for example, intervention coherence and burden. Data will also be sought on experiences relating to the research process, barriers to engagement, and trial and intervention feasibility. Interviews with young women will additionally examine views about the minimum meaningful change in the THS, i.e., the smallest change on this scale that they would consider to reflect a meaningful improvement. Interviews will be conducted over video or telephone calls, or in-person, according to participants’ preferences.

### Sample size

The primary outcome for the definitive RCT is hope as measured with the 12-item self-report THS [15] and the primary endpoint is 16 weeks post-randomisation. To allow precise estimation of the SD of THS for checking the definitive RCT sample size, the feasibility trial sample size is 70, 35 per arm [61]. We will use the feasibility trial to sense-check a 0.4 effect size for powering the definitive trial stage by assessing the SD of THS and exploring the minimal important difference in hope with a subsample of young women. We will additionally collect qualitative interview data from approximately 10 young women and 10 mentors. The qualitative sample size will be finally determined using the information power principle [62], such that the final sample size will reflect the richness and comprehensiveness of the obtained data.

### Recruitment

We will recruit young women participants via three main routes. First, we will engage with local authorities, charities, and voluntary sector services spanning youth work, employability services, education, social care, care experience, substance use, and non-NHS mental and sexual health services. These organisations will be identified using regular scoping of youth provision within the study regions, coupled with snowballing strategies and as guided by the PIP and existing referrers. We will work with local services to disseminate recruitment materials, maintaining a regular physical researcher presence in team meetings and drop-in services, and joining meetings between young women and their supporting services where appropriate. Organisations will be asked to first gather permission from potentially eligible young woman before then giving their contact details to the research team.

Second, we will engage with NHS providers to promote the project through GP practices and community pharmacies. These primary care organisations will be asked to display study posters in waiting areas and promote the study to potentially eligible young women during usual clinical contacts and/or remotely (e.g., by text message).

Third, we will invite young women to self-refer, by promoting the project in relevant physical spaces, for example placing posters in community centres and hubs, libraries, and food banks. We will also promote the project online through a dedicated Instagram account (@looking_forward_project). We will also use a limited amount of paid social media advertising in which the study flyer is “boosted” to non-followers within the study age range and relevant geographical areas.

Mentors will either be nominated by young women themselves (a youth-initiated mentor) or be allocated by the research team. Study-allocated mentors will be selected from a pool of relevant professionals and community volunteers. The former group will comprise professionals from referring organisations, mentor supervisors, and additional professionals from relevant local networks who express an interest in the study. The latter group will be identified through promotion of the study to local groups, organisations, and institutions deemed relevant and appropriate, such as local universities, women’s groups, retired people, and the emerging graduate psychology workforce.

### Data collection methods and participant timeline

#### Referral and consent

All referrals will be screened initially for appropriateness. This may involve asking an external referrer or self-referring individual for more information, for example if their geographical location is not provided at point of referral. Care will be taken to try and identify potential “impostor” participants, i.e., individuals falsely purporting to be an eligible young woman. “Impostor” participation is a growing problem and can be challenging to identify [63]. Researchers will be vigilant for invalid contact details, inconsistency (for example, in reported age or location) and contradictory responses [63, 64], and may ask to see identification to confirm date of birth and/or residence if needed [63, 64].

Research consent will be invited using a participant information sheet (PIS) and informed consent form (see Additional files 1 and 2). Research consent will be also sought separately from a mentor following allocation of an index participant to the HOPEFUL Together arm. The PIS (including easy-read and video formats) will be emailed and/or posted to the prospective participant at least 24 h before inviting consent. The consent meeting will involve a discussion about the nature and objectives of the trial and possible risks and benefits associated with participation. The prospective participant may invite another party, for example a parent or carer, to be present at this meeting should they wish. If there is any concern regarding capacity to provide informed consent, the researcher will not invite consent at that time.

Participants will be encouraged to engage and remain in the study with flexible and assertive strategies, including flexible meeting locations and modes of assessment completion. Prospective participants will be advised that participation is voluntary and that they can withdraw at any time. In the case of declined consent or later withdrawal, participants will be invited to provide reasons for their decision if they would like to do so.

#### Eligibility and baseline assessment

Following consent from a young woman, they will be asked to complete the eligibility assessment (Table 3) in an in-person or video or phone call with a researcher. Following completion, the researcher will conduct an eligibility assessment review with a senior member of the trial team. Participants deemed ineligible may be rescreened if their reason for ineligibility is subject to change, for example, current EET activity that is due to end.

**Table 3:** Schedule of procedures

Procedures	Visits/assessment points				
	Consent/eligibility	Baseline	Intervention	16-week follow-up	12-month follow-up
Informed consent	*X*				
Eligibility (screening) assessment (listed in order of completion)					
*Demographics part one*	*X*				
*Time Use Survey (TUS)*	*X*			*X*	*X*
*Patient Health Questionnaire (PHQ-9)*	*X*			*X*	*X*
*Demographics part two*					
Baseline assessment (measures listed in order of completion)					
*Trait Hope Scale (THS)*		*X (young women and mentors)*		*X (young women and mentors)*	*X*
*Short Warwick-Edinburgh Mental Well-Being Scale (SWEMWBS)*		*X (young women and mentors)*		*X (young women and mentors)*	*X*
*Generalised Anxiety Disorder Scale (GAD-7)*		*X*		*X*	*X*
*UCLA Loneliness Scale (UCLA-8)*		*X*		*X*	*X*
*General Help-Seeking Questionnaire (GHSQ)*		*X*		*X*	*X*
*Morris IV revision of logical memory test*		*X*			
*Meaning in Life self-report scale (MLQ)*		*X*		*X*	*X*
*Controlled Oral Word Association Test (COWAT)*		*X*			
*Combined Social Interaction Anxiety Scale short form (SIAS-6) and Social Phobia Scale short form (SPS-6)*		*X*		*X*	*X*
*Social and Occupational Functioning Scale (SOFAS)*		*X*		*X*	*X*
*Client Service Receipt Inventory (CSRI)*		*X*		*X*	*X*
Randomisation		X			
Peri-intervention assessments					
*Working Alliance Inventory- Short Revised (WAI-SR)*			*HOPEFUL TOGETHER ONLY, c. intervention session 3, completed by young women and mentors*		
*Goal-Based Outcome Tool (GBOT)*			*HOPEFUL TOGETHER ONLY*		
*Adherence data*			*HOPEFUL TOGETHER ONLY, completed by mentors*		
Adverse events/effects					
*Adverse Events Checklist*				*X*	*X*
*Modified Edinburgh Adverse Effects of Psychological Therapy Scale (EDAPTS)*				*Completed by young women and mentors*	
*Routine monitoring of adverse events*	*By researchers/mentor supervisors based on participant contacts*	*By researchers/mentor supervisors based on participant contacts*	*By researchers/mentor supervisors based on participant contacts*	*By researchers/mentor supervisors based on participant contacts*	*By researchers/mentor supervisors based on participant contacts*
Qualitative interview				*Completed by a subset of young women and mentors*	*Completed by a subset of young women and mentors*

Assessments completed by (or assessor-rated in relation to) young women unless otherwise specified

Following confirmation of eligibility, the baseline assessment will be completed in an in-person, video, or phone meeting with a researcher. This assessment comprises both observer-rated and self-report measures and the latter can be completed independently online, if preferred. In addition to the baseline assessment measures (see Table 3), a researcher will engage the young woman in the mentor selection process (see the “Interventions” section). We aim to complete the baseline assessment within a maximum 1 month of completing the eligibility assessment.

#### Intervention adherence and adverse effects

The assessment of intervention adherence (HOPEFUL Together arm) will be completed by an unblind research team member shortly before the 16-week post-intervention follow-up. Mentors will be asked to record details of their contacts with young women and the delivery of intervention components. These data will be supplemented by additionally asking young women about the completion of intervention components. Young women in the intervention arm will also complete the WAI-SR after about three mentoring sessions, and the EDAPT shortly before their 16-week post-intervention follow-up, by the trial manager (TM) or other unblinded trial team members. Mentors will be asked to complete the WAI-SR and EDAPT at the same approximate timepoints.

#### Follow-up outcome assessments

Follow-up outcome assessments will be conducted by a blind assessor at 16 weeks and 12 months post-randomisation (see Table 3). The assessments may be conducted with the support of a researcher in-person, by phone or video call, and/or via an individual link to an online REDCap survey. If a young woman is uncontactable, efforts to contact them and obtain follow-up data will continue periodically unless the young woman declines contact, withdraws, or the trial ends.

Adverse events (AEs) will be elicited in both arms at the 16-week and 12-month endpoints using a self-reported AE checklist administered to young women. Mentors complete the THS and SWEMWBS at baseline and 16-week follow-up with the support of the TM or another unblinded researcher. Young women will be offered a £20 shopping voucher after completing each assessment point. An additional £20 shopping voucher will be offered to young women and mentors who complete a qualitative interview.

### Randomisation and allocation

Randomisation will be independently implemented by the Brighton and Sussex Clinical Trials Unit (CTU) and stratified by relevant local authority area and age (16 to 18, 19 to 25 years) with permuted blocks of randomly varying lengths. Randomisation will use a 1:1 allocation ratio.

Following completion of the baseline assessment, the TM or other unblinded senior research team member will randomise the participant within REDCap. Following randomisation, the TM will contact the young woman by telephone to inform them of the randomisation outcome and then send a confirmation letter to the young woman and their GP.

After completion of their final follow-up, young women participants will be asked about intervention exposure to determine any presence and source(s) of contamination. Contamination is defined as access to the HOPEFUL intervention package, before their final follow-up assessment, for those allocated to the HOPEFUL Future arm. A contamination flag will be added to participants as relevant, and a sensitivity analysis produced accordingly. Further information is provided in the statistical analysis plan (SAP; Additional file 3).

### Blinding

Outcome assessors and the senior statistician (Bremner), but not participants, will be blind to intervention allocation. The trial statistician (Eddy) was unblinded prior to commencing the trial, at the point of signing off the full SAP (Additional file 3). Strategies to maintain the blind will include concealing allocation from blind staff, prohibiting discussions about allocation and intervention delivery, separate physical and digital storage of all blinded data, and prohibition of blind and non-blind trial staff from working in close physical proximity. If blind breaks occur, wherever possible, the blind will be reinstated by replacing the outcome assessor with another blind trial researcher. All breaks to the blind will be recorded and reported transparently.

### Statistical methods

The results from the feasibility trial will be reported in line with the Consolidated Standards of Reporting Trials (CONSORT) 2010 extension for pilot and feasibility trials [65]. Means (standard deviations) and ranges will be calculated for continuous normally distributed variables, and medians (interquartile ranges) and ranges for non-normally distributed continuous variables. Categorical variables will be summarised by frequencies and percentages. The amount of missing data will be reported. We will descriptively analyse CSRI data, characterising what constitutes usual support and how this may vary by area or arm. Further details are available in the SAP (Additional file 3).

We will estimate the SD of the THS (primary outcome) in young women. We will additionally use sense-check a 0.4 effect size or otherwise derive a proposed minimum meaningful change in the THS. Both the SD and the minimum meaningful change (if applicable) may be used to identify a needed update to the sample size calculation for the definitive trial.

Progression to the definitive RCT will be decided using performance against the progression criteria (see Table 2). If green criteria are met, progression to the definitive RCT will occur with no or minor changes, for example, amending the order of assessment questionnaires or wording of instructions. If amber criteria are met, progression to the definitive RCT will occur with non-substantial changes, for example, amending entry criteria or intervention components. If the progression criteria are met with no major changes needed, then we will incorporate the feasibility trial into the definitive trial. If neither green nor amber criteria are met, with the agreement of our Trial Steering Committee (TSC) and Data Monitoring and Ethics Committee (DMEC) committees, the trial will end as a standalone feasibility trial.

### Qualitative methods and integration

Qualitative data will be analysed using a thematic analysis [66] approach that aims to involve multiple perspectives in the sample and the analysis team. Deductive analysis will be used to identify markers of intervention acceptability, such as intervention coherence and burden [60], and specific recommendations for protocol amendments. Inductive analysis will be used to identify additional experiences relevant to the research questions [67]. We aim to sample young women and mentors with differing patterns and experiences of intervention engagement, and to include young women from both trial arms. The analysis will involve both peer and non-peer researchers to enable deeper understanding and more nuanced reporting, whilst keeping the voices of young women at the centre.

Finally, a mixed method matrixing approach [68] will be used to integrate quantitative and qualitative data pertaining to each research question. We will work with our PIP and oversight groups to confirm necessity and sufficiency of suggested protocol changes. Intervention amendments, as necessary, will be reflected in a refined Theory of Change.

### Adverse events (harms)

An AE is an untoward medical or psychological occurrence, which becomes a serious adverse event (SAE) if meeting any seriousness criterion, for example being life-threatening or requiring crisis care. We pre-empted the following as “expected” AEs in that they are relatively common to NEET young women: distress, self-harm, suicidal ideation, drug, and alcohol use.

AE may be identified through structured assessments, e.g., the AE checklist (both trial arms) and EDAPTS (HOPEFUL Together arm only; completed by young women and mentors), and spontaneous disclosures made by research participants or others (e.g., mentors, referrers). All AEs will be recorded in detail and with reference to the relatedness, expectedness, and seriousness of the event. Any potentially serious and/or related events will be reported to the co-leads (or delegate) as soon as possible for review. SAEs will be reported to the sponsor by the next working day after the co-leads become aware of the event. If an SAE is identified as related to the trial and unexpected, it would be reported as a Serious and Unexpected Adverse Reaction (SUSAR) to the NHS REC within 15 days of the co-leads becoming aware of the event. AEs will be reported to the DMEC at each meeting, including the number of the events overall and split by trial arm, with further detail on the nature of all related and serious events.

## Public involvement

We aim to foreground the voices of NEET young women and their supporters throughout the research lifecycle. Fountain, who has personal and professional experience of supporting NEET young people, reprises her public involvement lead role from the previous developmental study [14]. We have convened a Public Involvement Panel (PIP) of eight young women with recent experience of being NEET, co-ordinated by a facilitator who is herself a young woman with relevant lived experience. In the pre-funding period, this panel met to review documents drafted for submission to ethical review. The PIP will meet on an ongoing basis throughout the trial (c. six monthly). Later meetings will involve reviewing protocol amendments and informing recruitment, retention, and dissemination strategies. The TSC will include two public members with recent experience of being a NEET young woman and/or supporting NEET young women. Involvement activities and outcomes will be logged and reported, in keeping with the GRIPP2 framework [69]. We will create opportunities for colleagues with lived experience to be involved in creating and disseminating trial outputs. We will additionally employ young women with experience of being NEET as peer researchers, with responsibility for collecting qualitative interview data and contributing to qualitative analysis and publication-writing.

## Roles and responsibilities

### Protocol development

Berry led the development and drafting of the protocol with co-lead investigator Michelson. Protocol contributions were made by Fountain, Wilson, Forbes, Bremner, and McCrone. Berry and Michelson also led on acquisition of funding, with co-investigators Fountain, Wilson, Forbes, and Bremner. Rawlinson and Bird contributed to protocol amendments and Eddy to the development of the statistical analysis plan (SAP).

### Trial and data management

The implementation of the trial protocol will be co-ordinated by a trial manager (TM) (Rawlinson) under the direction of Berry and Michelson. Research assistants (RAs) will be employed within each project locality to recruit and assess participants, and within collaborating universities (for example to assist with economic analysis). Those involved in project and intervention delivery will meet weekly to fortnightly in blind and unblinded operational meetings. The co-investigators and TM will meet monthly as the trial management group (TMG).

Berry, Michelson, and Rawlinson worked closely with Bremner, Eddy, and the Brighton and Sussex CTU to set up the trial REDCap database and develop the SAP. All relevant staff are trained in data collection and entry procedures by Berry and Rawlinson. REDCap uses in-built protections such as pre-set permissible values and records an audit trail of data entered or amended. Access to the full dataset will be provided by Brighton and Sussex CTU to those involved in statistical and economic analyses. Qualitative interview data will be stored separately to REDCap in audio files (deleted once transcribed and checked) and typed transcripts. Following analysis and publication of trial outcomes, an anonymised derived version of the full trial dataset, with a data dictionary, will be made publicly available.

### Trial oversight

The oversight groups are the Data Monitoring and Ethics Committee (DMEC), Trial Steering Committee (TSC), and the PIP. Each group will meet approximately every 6 months. Within each meeting cycle, the PIP and DMEC will meet about 2 weeks before the TSC so that the latter can be informed by their recommendations.

The DMEC comprises one independent statistician and two independent clinical academics. The purpose is to oversee the safe and ethical conduct of the trial, including reviewing adverse events and effects. The TSC comprises ten members, two of whom are public members, reflecting expertise in mental health, women’s health, public health, trial and process evaluation methods, health economics, medical statistics, and experiences of being and supporting vulnerable young women. The purpose of the TSC is to assure the scientific integrity of the trial. Further details about membership of the DMEC and TSC are presented in the full research protocol approved by the Health Regulatory Authority, available on request.

## Ethics and dissemination

### Research ethics approval and consent

Ethical approval for the feasibility and definitive trial stages has been obtained from the Health Research Authority (24/LO/0521). The study sponsor is the University of Sussex. Participants will provide informed consent in writing before undertaking any research activities. The current version of the protocol is V3.0 (18/12/2025). Any subsequent amendments will be promptly communicated to relevant individuals and the funder.

### Confidentiality

We will collect only the minimal personal data needed for the trial to effectively run. Personal data will not be entered into REDCap but will be stored, separately to research data, in locked filing cabinets in protected study sites and secure university or NHS computer systems. All research data will be archived and then destroyed after 10 years post-publication, with an anonymised derived version of the final data available on a public data sharing site. All personal information will be destroyed within 2 years of the study end date.

### Equality, diversity, and inclusion

We will work in line with the NIHR Equality, Diversity, and Inclusion (EDI) strategy [70] to embed diversity and inclusion considerations throughout the trial. We will use inclusive entry criteria with respect to age and the operationalisation of “coastal” and “woman”—with the latter focusing on self-identification. We aim to promote access through “easy read” and video informational materials and have funds to translate these materials into multiple languages. We will use a gentle yet proactive method of participant engagement to “reach-in” to withdrawn and marginalised communities, who often experience long-term patterns of social withdrawal and feel distrustful of health and education providers. We will offer flexible research meetings and assessments and funds for travel or other associated costs (e.g. mobile phone data) as needed to enable participant/public member involvement. We will reflect on issues pertaining to equality, diversity, and inclusivity with our PIP.

## Discussion

This feasibility trial will test the feasibility and acceptability of conducting a randomised controlled trial (RCT) to evaluate a mentor-supported, hope-focused intervention (HOPEFUL). The feasibility phase will examine the extent to which it is possible to recruit and retain, with complete data, NEET young women and mentors (who may be either “youth-initiated” or “study-allocated”) in a randomised controlled trial of HOPEFUL plus usual support versus usual support alone. We will additionally seek to understand trial and intervention experiences of young women and mentors and to identify any necessary refinements to the RCT design and/or intervention specification. Subject to feasibility trial findings, we will progress to a definitive trial. If warranted, the feasibility trial outcome data will be incorporated into the larger trial, functioning as an internal feasibility pilot.

We will disseminate findings using tailored outputs for participants, the public, academics, health and social care practitioners and commissioners, and policymakers. In the longer term, we anticipate practice impacts through scaled-up access to HOPEFUL. The ultimate goal of this programme of work is to improve the mental health and social outcomes of a vulnerable group of young women and to strengthen their networks, with associated economic and societal gains.

## Supplementary Information

Supplementary Material 1.

Supplementary Material 2.

Supplementary Material 3.

Supplementary Material 4.

Supplementary Material 5.

## Data Availability

Data sharing is not applicable to this article as no datasets were generated or analysed during the current study.

## Abbreviations

AE: Adverse event
AR: Adverse reaction
COWAT: Controlled Oral Word Association Test
C-SSRS: Columbia-Suicide Severity Rating Scale
CSRI: Client Service Receipt Inventory
CTU: Clinical trials unit
DMEC: Data Monitoring and Ethics Committee
EDAPTS: Edinburgh Adverse Effects of Psychological Therapy Scale
EDI: Equality, diversity, and inclusion
EET: Education, employment, and training
GAD-7: Generalised Anxiety Disorder scale
GBOT: Goal-Based Outcome Tool
GHSQ: General Help-Seeking Questionnaire
GP: General practitioner
MLQ: Meaning in Life Questionnaire
NEET: Not in education, employment, and training
NHS: National Health Service
PHQ-9: Patient Health Questionnaire
PIP: Public Involvement Panel
PIS: Participant information sheet
RA: Research assistant
RCT: Randomised controlled trial
SAE: Serious adverse event
SAP: Statistical analysis plan
SAR: Serious adverse reaction
SD: Standard deviation
SIAS-6: Social Interaction Anxiety Scale short form
SOFAS: Social and Occupational Functioning Scale
SOPs: Standard operating procedures
SPS-6: Social Phobia Scale short form
SUSAR: Suspected Unexpected Serious Adverse Reaction
SWEMWBS: Short Warwick-Edinburgh Mental Well-Being Scale
THS: Trait Hope Scale
TiDIER: Template for intervention description and replication
TM: Trial manager
TMG: Trial management group
TSC: Trial Steering Committee
TUS: Time Use Survey
UCLA-8: UCLA Loneliness Scale, short form
WAI-SR: Working Alliance Inventory, short revised form
