# Supplementary material for: A pragmatic adaptive trial of hope-focused mentoring to improve mental health and social outcomes for young women who are not in education, employment or training in deprived coastal areas (The Looking Forward Project): feasibility trial stage protocol
Source: Pilot Feasibility Stud. 2026 May 30;12:105. doi: 10.1186/s40814-026-01852-4 (PMC13425958; doi:10.1186/s40814-026-01852-4)
Supplement: Supplementary file 1 — Supplementary Material 1. [file 40814_2026_1852_MOESM1_ESM.docx]

**Participant Information Sheet for Young Women**

**The Looking Forward Project**


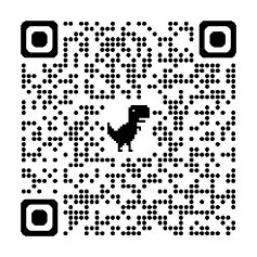
We would like to invite you to take part in a research study called The Looking Forward Project. Before you decide to take part, we would like you to understand why this research is being done and what it will involve for you. Taking part is completely your choice.

Please take as much time as you need to read this information sheet. A researcher will also go through it with you and will be happy to answer any questions. You can watch a video version of this information sheet by scanning the QR code or using this [link](https://youtu.be/GiouDA2aKE4).

**Summary**

- The Looking Forward Project is for young women aged 16-25 years who are Not in Education, Employment, or Training (NEET).
- The project is a research study testing a new public health programme for young women who are NEET called HOPEFUL. The programme involves a package of activities and videos with the goal to help young women feel more hopeful.
- To test whether the programme is helpful, **half of the people taking part will receive HOPEFUL straight away and will be supported by a mentor**. **The other half of people will continue with their usual care and then get the HOPEFUL package after 12 months to use however they like**.
- Which version of the programme people get is decided randomly by a computer with a 50/50 chance, like flipping a coin. We will then assess how the two groups get on after 4 months and again after 12 months.
- If you are interested in taking part, you will first meet with a researcher to decide together whether the project is right for you. Taking part is your choice and it’s okay if you do not want to.
- If you take part, you will meet a researcher at the start of the study to do an assessment. You will then be asked to do further assessments after 4 months and 12 months. To thank you for your time, will get a £20 voucher after each assessment (worth up to £60 in total).

**If you are interested, please read on for more information about the Looking Forward Project.**

## What is the aim of the Looking Forward Project?

We know that mental health and social problems are more common for young people who are not in education, employment, or training. We use “NEET” for short. These problems seem to be worse for young women who are NEET than men. These problems also seem to be worse for young people living in coastal areas, which often have fewer opportunities and less support.

The Looking Forward Project is testing a new public health programme, called “HOPEFUL”, for young women who are NEET and living in or near coastal areas. The programme aims to help young women feel more hopeful, which means believing in themselves and their ability to work towards their goals. Research shows that hope is important for mental health, wellbeing, and achieving our goals.

The HOPEFUL programme was created with NEET young women and the people that support them. It includes a package of activities and videos to help with developing a more hopeful mindset, identifying future goals, and making positive plans.

To understand the effects of HOPEFUL, we need to conduct a kind of test called a randomised controlled trial, or “trial” for short. This involves comparing two versions of the programme over time. In this project:

- **Half of the people taking part will receive HOPEFUL straight away and will get support from a mentor**. The mentor could be a trusted adult whom the young person already knows. If it not possible to find a known person to act as a mentor, the research team can offer an independent youth worker/psychologist.
- **The other half of people taking part will continue with any care or support they already receive and then get the HOPEFUL package after 12 months, to use however they like**. This could be used on their own or with a mentor they arrange for themselves.

## Who can take part?

This project is for young woman who are:

- Aged 16-25 years old
- Currently NEET
- Living in Sussex, Kent or East Anglia

If you agree to take part, we will first ask you some questions to check the project is right for you at this time.

## Do I have to take part?

No, you do not have to take part. It is completely up to you to decide whether you want to take part or not. You can take your time to think about it. If you do decide to take part, you can change your mind at any time, without giving a reason. Deciding not to take part, or to stop taking part at any point, will not affect any usual support you may be receiving or your medical or legal rights in any way.

## What will happen if I take part?

Here is an overview of what will happen if you take part in the Looking Forward Project.

**Eligibility meeting**

You will meet with a researcher to go through this information sheet and discuss any questions. If you decide to take part, you will be asked to complete a consent form. You will then be asked some more questions about your employment/education and mental health to check the project is right for you. We may ask to see (but not save or store) identification (ID) showing your name, photo, or address. We expect this meeting to take up to 1 hour in total.

**Baseline assessment**

If the project is right for you, you will be asked questions about how hopeful you are feeling, and about your wellbeing, mental health and relationships. There will also be practical tasks related to solving problems. We expect this to take about 45 minutes. A £20 shopping voucher will be given on completion.

**Random allocation**

A computer will randomly allocate you to one of the two groups described below. Random allocation means decided completely at chance - like tossing a coin. A researcher will tell you the outcome of the random allocation as soon as they can.

**Follow-up assessments**

After 4 months and 12 months, a researcher will invite you to complete the same questionnaires you did at baseline. Additional questions will ask about your experience of HOPEFUL (where relevant). In both groups, we will ask about health issues/social challenges you might have experienced as well as use of services. You can complete most of these online using a survey link in your own time, but a researcher can support you to do these in person if you prefer. A £20 voucher will be given after each assessment.


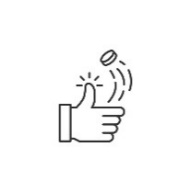


**HOPEFUL Future Group**

You will continue to receive any support you were already receiving. You will also get a list of other ways of accessing support. When the study ends, you will be given access to the HOPEFUL package to use however you like. You could use this by yourself or with a mentor that you arrange yourself.

**HOPEFUL Together Group**

You will be given access to a HOPEFUL workbook, on paper and online. You will start working through HOPEFUL with the 1:1 support of a mentor. We suggest you meet with your mentor regularly, for roughly 4-12 meetings over about 16 weeks. You can complete activities on your own too.

Some young women (and some mentors) will also be invited to take part in an optional individual interview, either after the 4-month or 12-month assessment. This interview will take about an hour and ask more in-depth questions about the experience of taking part in the study. Those who complete the interviews will get a further £20 shopping voucher as a thank you.

## How will I meet with the research team?

You can meet with the research team on days and at times that are suitable for you. You can meet with them either in-person, online or over the phone. If you meet online, this will be through video software (such as Zoom or Microsoft Teams). You will be asked to complete most questionnaires online using a survey link. However, the researcher can support you with this if you need, for example by reading the documents aloud and recording answers on your behalf. You will be able to take a break or skip past any question you would prefer not to answer. The meetings can be split into multiple shorter sessions if you prefer.

## Who are the mentors in HOPEFUL Together?

All young women will be asked to think about a trusted adult in their life who they might like to be their mentor and support them to use HOPEFUL. This could be someone you know personally or a professional who supports you elsewhere. If you are allocated to the HOPEFUL Together group, the project team will help to invite your chosen mentor to join the project. This mentor will be asked complete their own consent form. If you can’t think of a mentor who is able to take part, the project team will connect you with someone who can become your mentor. This could be someone like a youth worker, psychologist, a a local university student, or someone with experience of being NEET themselves. All mentors will get training in the HOPEFUL programme and receive support from a suitably experienced professional.

## What are the risks or downsides of taking part?

This project will involve answering questions about your mental health and wellbeing during assessments. Some people have told us that they have found it interesting and helpful to answer such questions. Some questions ask about topics that might feel sensitive, for example, about low mood and suicidal thoughts. You do not have to answer any questions that you do not want to answer. You are free to ask the researcher to move on or to stop the assessment altogether if you are finding it upsetting. If you become distressed, a researcher will provide details of support services and can help you to contact them.

## What are the benefits of taking part?

By taking part in this project, you will be contributing to research to learn whether and how HOPEFUL is helpful for young women who are NEET. You will also be offered £20 shopping vouchers as a thank you for your time at the three assessment timepoints (£60 total over 12 months) with another £20 available for taking part in a further interview.

## Will my involvement in the project be kept confidential?

The only people that will know you are taking part in this project will be the research team, your GP, the service that told us you may be interested (if there was one), the person who supervises your mentor if you are randomised to HOPEFUL Together, and anyone else that you choose to tell. We will inform your GP that you are taking part and which group you have been allocated to (i.e., HOPEFUL Together or HOPEFUL Future). We will also tell your GP when your involvement with the project comes to an end.

For young people in the HOPEFUL Together group, mentor supervisors will be told some information about the needs you might have, such as having an Education, Health and Care (EHC) plan, mental health problem diagnosis, special educational needs, or experience of care. This information will only be used to make sure that your mentor can be provided with the right type of supervision. Organisations employing mentor supervisors will sign a data protection agreement to make sure that they do not share this information.

Information collected about you in this project will be kept strictly confidential. We will only break this confidentiality where there are significant concerns about your safety or someone else’s safety. We will take all possible steps to discuss this with you first before contacting any relevant professional.

## How will you use information about me?

We will need to use information obtained from you for this research project. This information will include your name, contact details, and your GP details. The researchers will access this information to contact you through the trial. Responsible members of university governance teams may also check your records to make sure the research is being done properly. Members of the governance or research team who do not need to know who you are will not be able to see your name or contact details. You will be given a code number at the start of the study and all your data will be linked to this number rather than your name.

We will keep all information about you safe and secure. Information will be stored on password-protected secure NHS or university computer systems, or in locked filing cabinets in secure departments. Any personal information we have about you (your name and contact details) will be securely and confidentially destroyed two years after the study end date. Our universities will securely destroy all anonymised research data (data that does not identify you personally) 10 years after publishing the main study report.

If you take part in an audio-recorded interview, we will pay a professional transcription service to type up the audio-recording word-for-word. This called a transcript. We will use a company that is registered with the University of Sussex to create these transcripts. We will ensure that any identifiable information (for example, names of people or places) mentioned in your recording is removed. We will then securely destroy the audio file and keep only the non-identifiable transcript. No-one would be able to work out who you are from saved research data.

## What are my choices about how my information is used?

You can stop being part of the study at any time, without giving a reason, but we will keep information about you that we already have. We need to manage your records in specific ways for the research to be reliable. This means that we won’t be able to let you see or change the data we hold about you. After the end of the study, we may also use your anonymous research data (that does not include your name or contact details) within future research. You can find out more about how we use your information by contacting a member of the research team or at <https://www.hra.nhs.uk/planning-and-improving-research/policies-standards-legislation/data-protection-and-information-governance/gdpr-guidance/templates/template-wording-for-generic-information-document/>

## What will happen to the results of the project?

We will use the results of this project to learn whether and how the HOPEFUL programme is helpful for young women. Written reports of the results will be presented in scientific journals, at conferences, and to

people who provide services for young people. We will write a report of the results for participants, including a short video that we can send to you if you wish. We will also provide information about the results on our project website. It will not be possible to identify anyone who has taken part in the study from any of the reports or videos.

## Who is organising and paying for the research?

The research is being run by a team of researchers from the Brighton and Sussex Medical School, King’s College London, University of Kent, Sussex Partnership NHS Foundation Trust, Norfolk & Suffolk NHS Foundation Trust, and University of Greenwich. The project is sponsored by the University of Sussex and is funded by the National Institute for Health and Care Research.

## Who has approved the project?

Research projects like this can only go ahead if they have been approved by an NHS Research Ethics Committee. This committee checks that the risks of the research have been kept to a minimum and that we have given you all the information you need to decide whether to take part. This project has been approved by the London - Surrey NHS Research Ethics Committee (Reference IRAS 327723).

## What happens if something goes wrong?

The University of Sussex has insurance in place to cover its legal liabilities in respect of this study. If you are unhappy or have a concern about any aspect of this project, you can ask to speak to a member of the research team on the contact details below who will do their best to help.

If you would like to speak to someone outside of the research team for more information or if something goes wrong, you can speak to the sponsor, University of Sussex, by emailing [researchsponsorship@sussex.ac.uk](mailto:researchsponsorship@sussex.ac.uk) (or asking someone else to do this on your behalf).

## Contact details:

If you would like more information or want to discuss anything about the project, you (or someone on your behalf) can get in touch with a member of the research team using the contact details below. They can typically be reached during office hours, Monday to Friday 9am to 5pm, and will do their best to get back to you as soon as possible.

Research assistant: Charlotte Rawlinson: [c.rawlinson2@bsms.ac.uk](mailto:c.rawlinson2@bsms.ac.uk)/hopeful@bsms.ac.uk (Sussex) /Arti Makwana: [a.p.makwana-37@kent.ac.uk](mailto:a.p.makwana-37@kent.ac.uk) (Kent) / Zoe Inman [zoe.inman@nsft.nhs.uk](mailto:zoe.inman@nsft.nhs.uk) (East Anglia)

Trial manager: Charlotte Rawlinson, Brighton and Sussex Medical School, University of Brighton
Email: [c.rawlinson2@bsms.ac.uk](mailto:c.rawlinson2@bsms.ac.uk)

Chief Investigators: Dr Clio Berry, Brighton and Sussex Medical School, University of Brighton
Email: [c.berry@bsms.ac.uk](mailto:c.berry@bsms.ac.uk)

Dr Daniel Michelson, Kings College London

Email: [daniel.michelson@kcl.ac.uk](mailto:daniel.michelson@kcl.ac.uk)
