# Supplementary material for: A pragmatic adaptive trial of hope-focused mentoring to improve mental health and social outcomes for young women who are not in education, employment or training in deprived coastal areas (The Looking Forward Project): feasibility trial stage protocol
Source: Pilot Feasibility Stud. 2026 May 30;12:105. doi: 10.1186/s40814-026-01852-4 (PMC13425958; doi:10.1186/s40814-026-01852-4)
Supplement: Supplementary file 2 — Supplementary Material 2. [file 40814_2026_1852_MOESM2_ESM.docx]

**
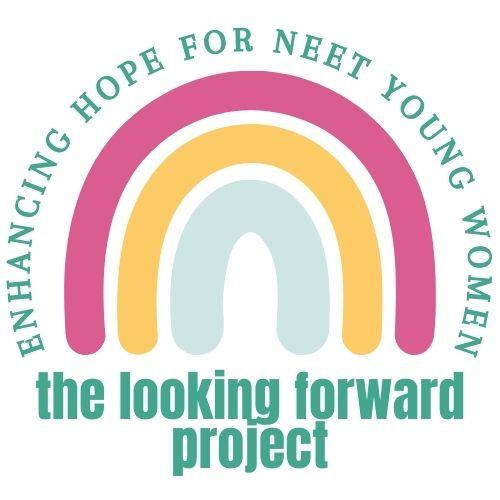
**

**Informed Consent Form for Young Women**

This form is for you to show that you understand and agree to take part in the Looking Forward project. Please read the list of statements below and if you agree with each one, please put your initials in the box. For you to take part in the project, we need to be sure that you agree (and feel comfortable with agreeing) with all the statements.

If you have any questions about the consent form, please contact the research team ([hopeful@bsms.ac.uk](mailto:hopeful@bsms.ac.uk) (Sussex), Arti [a.p.makwana-37@kent.ac.uk](mailto:a.p.makwana-37@kent.ac.uk) (Kent), or Zoe [zoe.inman@nsft.nhs.uk](mailto:zoe.inman@nsft.nhs.uk) (East Anglia)) before completing this form.

**If you agree with each statement, please put your initials in the box next to it:**

| 1. I confirm that I have read and understood the Participant Information Sheet (Version 3.1, date 07/08/2025) and have had the opportunity to have my questions answered. |  |
| --- | --- |
| 1. I understand that taking part is voluntary and that I am free to leave the project any time, without giving any reason, and without my health care or legal rights being affected. |  |
| 1. I understand that I can withdraw the data I have provided by contacting the research team to ask for my data to be withdrawn up to four weeks after I have provided the data. |  |
| 1. I understand that I may be asked to show identification displaying my name, photo, and/or address. I understand that the research team will not save or store my identification.  \|  \| \| --- \| |  |
| 1. I agree that the service who suggested that I take part (if this applies), my general practitioner (GP), and the person supervising my mentor (if I am randomised to HOPEFUL TOGETHER) will be informed that I am taking part in the project. I am happy for information about me to be shared between the research team and these services if the research team believe that there is a risk to my or someone else’s health or safety. |  |
| 1. I understand that if I take part in an interview as a part of my involvement in the project, my responses will be audio-recorded so that what I say will be accurately recorded. |  |
| 1. I understand that my personal information will be processed for this research study. I understand that such information will be treated as strictly confidential and handled in accordance with the UK Data Protection Legislation. Further information can be found in the Privacy Notice here: <https://www.sussex.ac.uk/about/website/privacy-and-cookies/privacy>. |  |
| 1. I agree that my anonymised information (where my name or identifying details will NOT be included), including word-for-word quotes of things I have said to the research team, can be used in reports, on the project website, and in further research projects. |  |
| 1. I understand that information collected about me during this project may be looked at by professionals responsible for ensuring the research is being carried out properly. I give permission for these individuals to have access to my information. |  |
| 1. I understand that if I am randomly allocated to HOPEFUL TOGETHER, the research team will share with individuals providing mentor supervision the following information: my name and whether I have an Education, Health and Care (EHC) plan, mental health problem diagnosis, special educational needs, or am a care leaver/ care experienced. I understand the purpose of this is to ensure the best supervisor can be found for my mentor. |  |
|  |  |
| 1. I agree to take part in The Looking Forward Project. |  |

SIGN/TYPE FULL NAME: _______________________________________

Date: ____________________
