# Supplementary material for: A pragmatic adaptive trial of hope-focused mentoring to improve mental health and social outcomes for young women who are not in education, employment or training in deprived coastal areas (The Looking Forward Project): feasibility trial stage protocol
Source: Pilot Feasibility Stud. 2026 May 30;12:105. doi: 10.1186/s40814-026-01852-4 (PMC13425958; doi:10.1186/s40814-026-01852-4)
Supplement: Supplementary file 3 — Supplementary Material 3. [file 40814_2026_1852_MOESM3_ESM.pdf]

The Looking Forward Project (TLFP)

A pragmatic adaptive trial of hope-focused mentoring to improve mental health and social outcomes for young women who are not in education, employment or training in deprived coastal areas

Registration number: ISRCTN52288029

Statistical Analysis Plan

Version: 1.0

Date: 30 Apr 2025

Produced using protocol version 1.4 [18 Dec 2024]

Contents

1. Preface 8
2. Introduction 8
2.1 Background and rationale 8
2.2 Trial setting 9
2.3 Study objectives and endpoints 10
3. Progression criteria 12
4. Study methods 15
4.1 Study design 15
4.2 Randomisation 18
4.3 Sample size 18
4.4 Statistical interim analyses 18
4.4.1 Interim analyses 18
4.4.2 Early stopping guidelines 18
4.5 Timing of outcome assessments 18
5. Statistical principles 24
5.1 Confidence intervals and p values 24
5.2 Adherence and protocol deviations 24
5.3 Analysis populations 24
6. Study population 25
6.1 Eligibility criteria for young women 25
6.1.1 Inclusion criteria 25
6.1.2 Exclusion criteria 25
6.2 Eligibility criteria for mentors 25
6.2.1 Inclusion criteria 25

6.3 Recruitment.. 25

6.4 Baseline participant characteristics 26

7. Dataset.. 27

8. Analysis 28

8.1 Analysis methods.. 28

8.2 Harms 29

9. Appendix 1 The Trait Hope Scale 33

10. Appendix 2 Young woman baseline characteristics table............................... 34

11. Appendix 3 Mentor baseline characteristics 38

12. Appendix 4 Adverse event and serious adverse event tables 44

13. References.. 45

List of tables

Table 1: Persons contributing to the analysis plan 6
Table 2: Approval signatures 6
Table 3: Statistical Analysis Plan amendments 6
Table 4: Objectives and endpoints 10
Table 5: Pre-specified progression criteria 13
Table 6: Schedule of procedures 19
Table 7: The Trait Hope Scale questionnaire items 33

List of figures

Figure 1: Draft CONSORT flow diagram 17

List of abbreviations

AEs - Adverse events 32
CSRI - Client Service Receipt Inventory 19
DMEC - Data Monitoring and Ethics Committee 9
EDAPTS - Edinburgh Adverse Effects of Psychological Therapy Scale 32
EET - education, employment, or training 28
GCP - Good Clinical Practice 27
ITT - Intention to treat 27
NEET - Not in education, employment, or training 11
RCT - Randomised Controlled Trial 11
SAP - Statistical Analysis Plan 11
SD - Standard deviation 21
SOPs - Standard Operating Procedures 27
THS - Trait Hope Scale 21
TLFP - The Looking Forward Project 18
TM - Trial manager 27
TSC - Trial Steering Committee 10

Table 1: Persons contributing to the analysis plan

Name	Role
Saskia Eddy	Trial Statistician
Stephen Bremner	Senior Statistician
Clio Berry	Co-Chief Investigator
Daniel Michelson	Co-Chief Investigator
Charlotte Rawlinson	Trial Manager

Table 2: Approval signatures

Name	Role	Signature	Date
Stephen Bremner	Senior Statistician		30Apr2025
Clio Berry	Co-Chief Investigator		30/04/2025
Daniel Michelson	Co-Chief Investigator		30.04.25

Table 3: Statistical Analysis Plan amendments

Version updated	Updated version number	Summary of changes	Author of changes	Date	Blinding status of person making the changes
0.1	0.2	Feedback from Stephen Bremner (SB) and Clio Berry (CB) incorporated, such as clarification of the endpoints, addition of the trial registration number and minor wording changes.	Saskia Eddy (SE)	15/01/24	SE - Blind
0.2	0.3	Feedback from Jessica Green (Data Monitoring and Ethics Committee (DMEC)) incorporated, such as clarification of adverse event reporting and minor wording changes.	SE	04/02/25	SE - Blind

Version updated	Updated version number	Summary of changes	Author of changes	Date	Blinding status of person making the changes
0.3	0.4	Minor typographical amendments	SE	19/02/25	SE - Blind
0.4	0.5	Feedback from Fiona Warren (Trial Steering Committee (TSC)) incorporated and SAP updated after protocol V1.4 released.	SE	03/03/25	SE - Blind
0.5	0.6	Minor typographical amendments.	CB	29/04/25	CB - Unblind
0.6	0.7	Minor typographical amendments.	SB	29/04/25	CB - Blind
0.7	1.0	Authorisation by study co-chief investigators CB, Daniel Michelson (DM), and senior statistician, SB.	CB, DM, SB	30/04/25	CB – Unblind, DM – Unblind, SB - Blind

1. Preface

This document is to provide a more technical and detailed description of the principal features of the analysis stated in the protocol, and to include detailed procedures for executing the statistical analysis of the primary and secondary variables and other data. This document was created following statistical analysis plan (SAP) specific reporting guidelines[1 2]. This SAP was reviewed by the trial management group, the independent DMEC statistician (30th January 2025 to 14th February 2025) and the independent TSC statistician (24th February 2025 to 11 April 2025). This SAP is for the Looking Forward Project feasibility trial and will be amended for the definitive trial, if the feasibility trial is found to be feasible. This SAP was created by SE while she was blinded. Once version 1.0 has been finalised i.e. signed by SB and co-CIs, SE will be unblinded. SB will remain blinded.

2. Introduction

2.1 Background and rationale

Young women who are not in education, employment, or training (NEET) are growing significantly in number. They have poorer mental health and social outcomes relative to young women who work and/or study and compared to NEET young men. Gender disparities are compounded in deprived coastal areas, in which NEET young women especially lack self-agency and aspirations. Research by our group [3 4]and others shows that greater hope reduces the risks of staying NEET and of having mental health problems. We have developed a hope-focused intervention (called HOPEFUL) for this group. HOPEFUL is a flexible modular programme, delivered over 4-12 weeks, for creating a hopeful mindset and learning skills in setting and pursuing personally meaningful goals. Support is provided by a youth-initiated mentor; someone that NEET young women select from their existing network.

An adaptive trial will be conducted to generate evidence on the feasibility, effectiveness, and cost-effectiveness of the HOPEFUL intervention. The specific objectives are to test the feasibility of conducting a randomised controlled trial (RCT) to evaluate HOPEFUL

and refine its design (feasibility trial) and then complete a definitive RCT with economic and process evaluations (definitive trial).

The primary feasibility research question is, is it feasible to recruit and retain NEET young women and mentors in a trial of HOPEFUL plus mentoring and usual support vs. usual support and access to the HOPEFUL materials at end of the trial, conducted in coastal and proximal local authorities in Sussex, Kent and Norfolk, with recruitment focused on deprived neighbourhoods).

The primary definitive RCT research question is, does HOPEFUL improve hope (primary outcome) and secondary outcomes of mental health symptoms, wellbeing, life meaning, time use, loneliness, and help-seeking of NEET young women at the primary endpoint of 16 weeks and secondary endpoint of 12 months post-randomisation compared to usual support services? Secondary research questions pertain to: intervention cost-effectiveness; how NEET young women and mentors experience HOPEFUL and its safety and acceptability; and mechanisms of intervention effects and contextual moderators.

2.2 Trial setting

The trial will run in Sussex, Kent and Norfolk, focusing on local authority areas containing a coastline or estuary as well as proximal local authority areas (i.e., within the same geographical county) that may not be directly situated on a coastline or estuary. This pragmatic definition reflects the fact that there is no established consensus for the term "coastal"[5] in public health, civic or demographic contexts.

2.3 Study objectives and endpoints

The objectives and their endpoints are described in Table 4.

Table 4: Objectives and endpoints

Overarching objective	Objective	Objective type	Endpoint	Estimator	Note
1. Test the feasibility and acceptability of conducting an RCT to evaluate HOPEFUL	1.1 Is it feasible to recruit and retain, with complete data, NEET young women and mentors in a randomised controlled trial of HOPEFUL plus usual support versus usual support alone?	Quantitative	**Recruitment:** Proportion of the target of NEET young women recruited within the specified recruitment period	$\frac{n_r}{70}$x100%	Where n_r is the total number of young women recruited during the recruitment period.
			**Data completeness:** participants provide primary outcome data at post-intervention assessment	$\frac{n_p}{n_{rand}}$x100%	Where n_p is the total number of participants that provide primary outcome data (12-item self-report Trait Hope Scale (THS)) at 16 weeks.

Overarching objective	Objective	Objective type	Endpoint	Estimator	Note
	1.2 Can HOPEFUL be delivered as intended and do NEET young women and mentors form positive mentoring relationships?	Qualitative			
	1.3 How do NEET young women and mentors experience trial participation and HOPEFUL?	Qualitative			
2. Refine the RCT design	2.1 What is the estimated standard deviation of the Trait Hope Scale (primary outcome) in NEET young women?	Quantitative	Estimated standard deviation of the Trait Hope Scale at 16 weeks post-randomisation	σ_{THS}	The estimated standard deviation may be used as a sense check of the effect size used in the definitive trial sample size calculation.
	2.2 What do NEET young women consider to be the minimum meaningful change in this measure of hope?	Qualitative	Young Women Interview	N/A	N/A
	2.3 What changes, if any, are needed to increase acceptability and feasibility of the intervention and research protocols?	Qualitative	Young Women Interview	N/A	N/A

3. Progression criteria

Progression to a definitive trial is determined based on a series of pre-specified progression criteria (Table 5). If green criteria are met, progression to the definitive RCT will occur with no or minor changes, e.g., amending assessment order to maximise engagement. If amber criteria are met, progression to the definitive RCT will occur with non-substantial changes, e.g., amending entry criteria or intervention components. If the progression criteria are met with no major changes needed, then (with agreement from the TSC and DMEC committees), the feasibility stage data will be incorporated into the definitive superiority effectiveness RCT. The remaining sample size and duration of the full effectiveness trial will be updated accordingly. If neither green nor amber criteria are met, with the agreement of the Trial TSC and DMEC committees, the project will end as a standalone feasibility trial.

Table 5: Pre-specified progression criteria

No.	Green	Amber	Red	Estimator	Timepoint	Notes
1	Recruitment of 100% (n=70) of the target of NEET young women within the specified recruitment period.	Recruitment of 50 to less than 100% (n=35-69) of the target of NEET young women within the specified recruitment period.	Recruitment of less than 50% (n≤34) of the target of NEET young women within the specified recruitment period.	$\frac{n_r}{70}$x100%		Where n_r is the total number of young women recruited (i.e. consented) during the recruitment period.
2	At least 40% of young people identified during the recruitment process are eligible and interested in participation	30 to less than 40% of young people identified during the recruitment process are eligible and interested in participation	Less than 30% of young people identified during the recruitment process are eligible and interested in participation	$\frac{n_{ei}}{n_i}$x100%		Where n_{ei} is total the number of young women eligible and interested in participation and n_i is the number identified during the recruitment process
3	At least 60% of NEET young women allocated to HOPEFUL complete 4 or more sessions	40 to less than 60% of young women allocated to HOPEFUL complete 4 or more session	Less than 40% of NEET young women allocated to HOPEFUL complete 4 or more sessions	$\frac{n_f}{n_h}$x100%		Where n_f is the total number of young women allocated to HOPEFUL and completing 4 or more

No.	Green	Amber	Red	Estimator	Timepoint	Notes
						sessions (the 4 sessions can be any sessions), and n_h is the total number of young women allocated to HOPEFUL.
4	At least 80% of participants provide primary outcome data at post- intervention assessment	50 to less than 80% of participants provide primary outcome data at post-intervention assessment	Less than 50% of participants provide primary outcome data at post-intervention assessment	$\frac{n_p}{n_{rand}}$ x100%	16 weeks	Where n_p is the total number of participants that provide primary outcome data (12-item self-report Trait Hope Scale (THS)) at 16 weeks. While n_{rand} is the total number of participants randomised. Please see Appendix 1 for the THS scoring details.
Note: No.=Number						

4. Study methods

4.1 Study design

The Looking Forward Project (TLFP) is an adaptive, assessor-blind, pragmatic, open label, parallel group superiority randomised controlled trial with 1:1 allocation to HOPEFUL with mentoring plus usual support (HOPEFUL TOGETHER) versus waitlist for HOPEFUL workbook plus usual support (HOPEFUL FUTURE). The programme consists of two stages, a feasibility trial, followed by a definitive trial. Unless otherwise indicated by pre-specified progression criteria, the feasibility trial will become an internal trial stage, and the feasibility sample will be subsumed into the definitive trial sample. The trial flowchart is shown in Figure 1.

HOPEFUL is a six-module psychosocial intervention comprising psychoeducation, cognitive, behavioural, and interpersonal activities. HOPEFUL is designed to be supported by a youth-initiated mentor. The intervention is delivered primarily 1:1 and in-person, supported by an accessible (non-patronising) online/paper workbook. The intervention comprises six modules, each of which contains core psychoeducational material and a menu of selectable activities to put newly learned concepts and skills into practice. The intervention could be delivered using formal or informal group-based approaches if wanted. The intervention has been designed to be delivered primarily in in-person meetings situated in non-stigmatising community locations. There is in-built encouragement in both young women's and mentor's materials to complete activities if wanted in appropriate outdoor places to increase physical activity and nature exposure, e.g., walking discussions with mentors. The intervention has been designed to be delivered over a flexible and collaboratively agreed number of sessions and spacing, with guidance of 4-12 sessions of 30-90 minutes each, spaced 3-14 days apart, over 4-12 weeks. A full dose is defined (as per the Theory of Change) as at least 4 sessions, with at least one each from modules 1, 2, and 4. The mentor's role is to provide supportive accountability, i.e., to provide encouragement to the NEET young woman to continue using the package and to offer assistance in understanding the components when needed. The mentor does not need to have or use specialist knowledge or technical

skills. The mentor is provided with brief, self-administered written and video-based training on hope, intervention model and components, and principles of providing supportive competency (with in-built self-rated competency tests), supervision (c. fortnightly), and a paper/digital intervention manual.

The comparator is usual support plus waitlist access to the HOPEFUL workbook for self-directed use. This arm is called HOPEFUL FUTURE. Following our experience in a previous RCT[6] involving young people with social disability and mental health problems, we anticipate that usual support will vary from nothing to support from social services, educational or employment services, and/or specialist mental health services. We aim to standardise this support by offering NEET young women a best practice support guide at allocation with information about local relevant provision. Data will be collected on usual support provision (form, frequency/duration), financial benefits, and informal/family support using the Client Service Receipt Inventory (CSRI [7]). At the end of their trial involvement (i.e., after their 12-month assessment and any subsequent qualitative interview to which they are invited), we will offer access to the HOPEFUL workbook to these participants for use however they choose. We will make no restriction as to whether young women could identify and request support from a mentor to use the intervention if they wanted to do so. However, the research team will not provide any support to identify or involve a mentor.

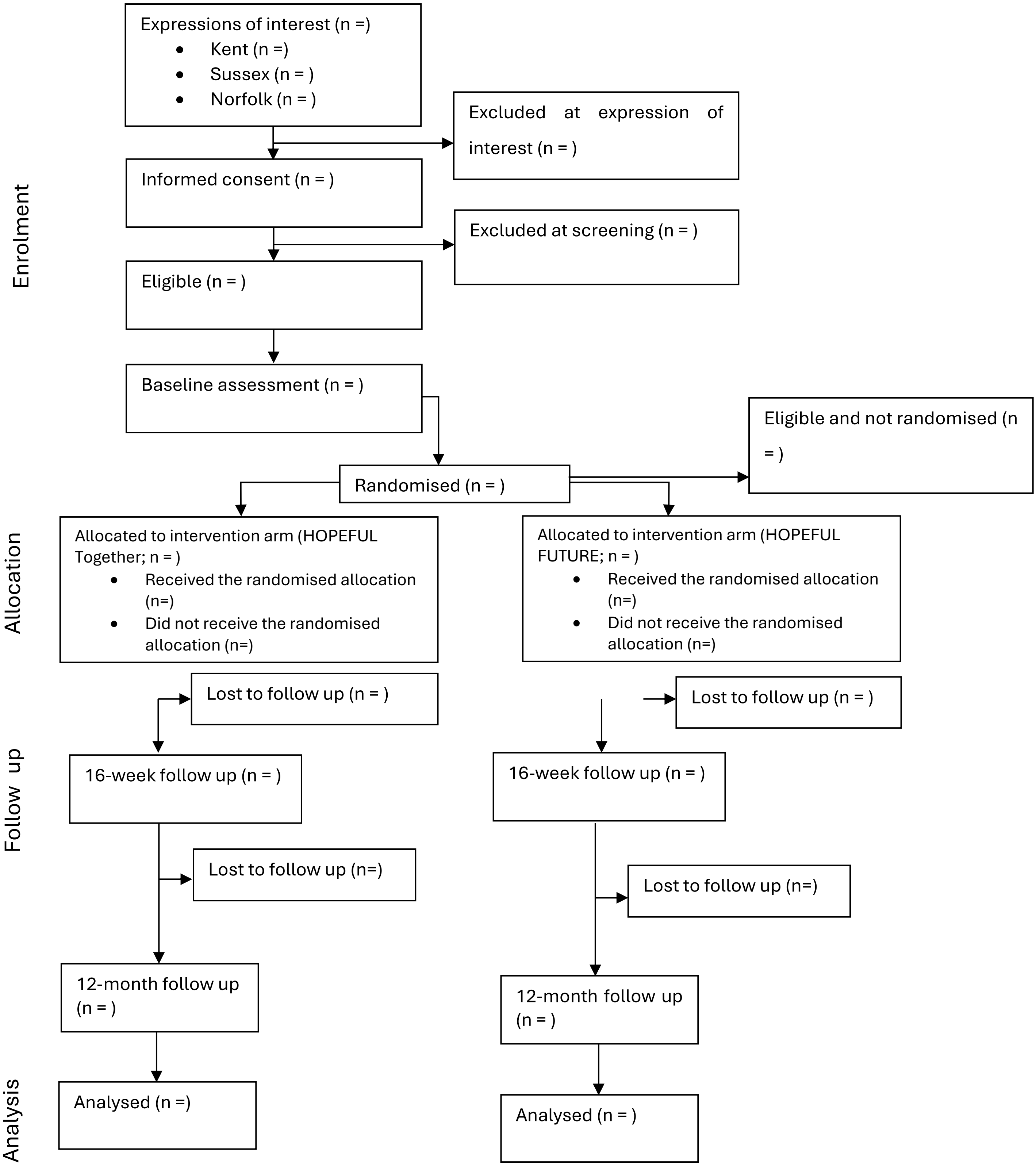

Figure 1: Draft CONSORT flow diagram

4.2 Randomisation

Randomisation is stratified by relevant local authority area and age (16 to 18, 19 to 25 years) with permuted blocks of randomly varying lengths. Randomisation will use a 1:1 allocation ratio. Patients are randomised to either HOPEFUL (intervention) or HOPEFUL-FUTURE (usual support plus waitlist access).

4.3 Sample size

The primary outcome for the definitive trial is hope as measured with the 12-item self-report Trait Hope Scale (THS)[8] at the primary endpoint of post-intervention (16-weeks). To allow precise estimation of the standard deviation (SD) of THS for checking the definitive RCT sample size, the feasibility trial sample size is 70, 35 per arm[9]

4.4 Statistical interim analyses

4.4.1 Interim analyses

There are no planned interim analyses for the feasibility trial.

4.4.2 Early stopping guidelines

The feasibility trial may stop early due to, amongst other things, safety concerns or external evidence.

4.5 Timing of outcome assessments

The schedule of study procedures is reported in the Table 6.

Table 6: Schedule of procedures

Procedures	Visits/ Assessment points				
	**Consent/ Eligibility**	**Baseline**	**Intervention**	**16 Week Follow Up**	**12 Month Follow Up**
Informed consent	X				
Eligibility (screening) assessment (listed in order of completion)					
Demographics part one	X				
Time Use Survey (TUS)	X			X	X
Patient Health Questionnaire (PHQ-9)	X			X	X
Demographics part two					
Baseline assessment (listed in order of completion)					

Procedures	Visits/ Assessment points				
	**Consent/ Eligibility**	**Baseline**	**Intervention**	**16 Week Follow Up**	**12 Month Follow Up**
Trait Hope Scale (THS)		X (Young women and mentors)		X (Young women and mentors)	X
Short Warwick-Edinburgh Mental Well-Being Scale (SWEMWBS)		X (Young women and mentors)		X (Young women and mentors)	X
Generalised Anxiety Disorder Scale (GAD-7)		X		X	X
UCLA Loneliness Scale (UCLA-8)		X		X	X
General Help-Seeking Questionnaire (GHSQ)		X		X	X
Morris IV revision of logical memory test		X			
Meaning in Life self-report scale (MLQ)		X		X	X

Procedures	Visits/ Assessment points				
	**Consent/ Eligibility**	**Baseline**	**Intervention**	**16 Week Follow Up**	**12 Month Follow Up**
Controlled Oral Word Association Test (COWAT)		X			
Combined Social Interaction Anxiety Scale short form (SIAS-6) and Social Phobia Scale short form (SPS-6)		X		X	X
Social and Occupational Functioning Scale (SOFAS)		X		X	X
Client Service Receipt Inventory (CSRI)		X		X	X
Randomisation		X			
Peri-intervention assessments					
Working Alliance Inventory- Short Revised (WAI-SR)			HOPEFUL TOGETHER ONLY, c. intervention session 3, completed		

Procedures	Visits/ Assessment points				
	**Consent/ Eligibility**	**Baseline**	**Intervention**	**16 Week Follow Up**	**12 Month Follow Up**
			by young women and mentors		
Goal-Based Outcome Tool (GBOT)			HOPEFUL TOGETHER ONLY		
Adherence data			HOPEFUL TOGETHER ONLY, completed by mentors		
Adverse events/effects					
Adverse Events Checklist				X	X
Modified Edinburgh Adverse Effects of Psychological Therapy Scale (EDAPTS)				Completed by young women and mentors	Completed by young women
Routine monitoring of adverse events	By researchers/mentor supervisors based on participant contacts	By researchers/mentor supervisors based on participant contacts	By researchers/mentor supervisors based on participant contacts	By researchers/mentor supervisors based on participant contacts	By researchers/mentor supervisors based on participant contacts

Procedures	Visits/ Assessment points				
	**Consent/ Eligibility**	**Baseline**	**Intervention**	**16 Week Follow Up**	**12 Month Follow Up**
Qualitative interview				Completed by a subset of young women and mentors	Completed by a subset of young women and mentors

5. Statistical principles

5.1 Confidence intervals and p values

Confidence intervals (95%) will be presented alongside estimates of potential effectiveness.

5.2 Adherence and protocol deviations

The assessment of intervention adherence pertains only to the HOPEFUL TOGETHER (HOPEFUL with mentor plus usual support) trial arm. Mentors will be asked to indicate the number of meetings they have with young women and will also be asked to indicate the modules and activities young women have completed. These data will allow us to describe how the intervention has been delivered and to identify young women who have received a "dose" of the intervention. A dose is defined as per the Theory of Change: at least four sessions, including one session each from modules 1, 2 and 4. The trial manager (TM) will monitor data completion on a regular basis and liaise with mentors to remind and/or support them to provide data.

The proportion of NEET young women allocated to HOPEFUL that complete 4 or more sessions will be calculated as a measure of adherence (Table 5).

A protocol deviation is defined as a change or departure from the protocol and/or GCP that does not result in harm to the study participants or significantly affect the integrity of the reported results. These could be logistical and/or administrative deviations from the protocol/Good Clinical Practice (GCP)/Standard Operating Procedures (SOPs) [10]. The deviations for the trial, will be provided to the trial statistician.

All significant protocol deviations will be summarised and reported to the TSC and DMEC.

5.3 Analysis populations

The intention to treat (ITT) population consists of all randomised participants, regardless of their compliance with the trial protocol. For example, whether the correct trial treatments were received and post-randomisation withdrawal. ITT will be the strategy of analysis for all outcomes. Adverse events will be reported according to ITT.

6. Study population

6.1 Eligibility criteria for young women

6.1.1 Inclusion criteria

1. Aged 16 to 25 years at time of consent
2. Identifying as a woman
3. NEET, operationalised as no involvement in education, employment, or training (EET) activity in past month as measured using the Time Use Survey – EET activity will not include informal activities such as casual babysitting, or one-off activities such as waiting tables at a single event
4. Resident in a local authority area in Sussex, Kent or Norfolk (consistent with definitions in Section 2.2)
5. Able to give informed consent

6.1.2 Exclusion criteria

1. Current EET activity (including being on temporary leave from and with planned return to their place of employment/education/training)
2. Serious risk of suicide, operationalised as a score of non-zero on the suicidality item of the Patient Health Questionnaire plus a rating of four or more out of seven with respect to severity of the suicidality

6.2 Eligibility criteria for mentors

There are no exclusion criteria for mentors.

6.2.1 Inclusion criteria

1. Aged 18 years or more at time of consent

2. Able to give informed consent

6.3 Recruitment

A CONSORT flow diagram (Figure 1) will be completed to summarise the number of young women that were:

- Assessed for eligibility (including the number eligible/ineligible*)

- Eligible and randomised
- Eligible and not randomised*
- Received the randomised allocation
- Did not receive the randomised allocation*
- Withdrawals*
- Lost to follow-up*
- Randomised and included in the analysis
- Randomised and excluded from the analysis*

*=including reasons

Additionally, the CONSORT diagram will include the number of expressions of interest from each local authority. A separate flow diagram for mentors displaying the following may be created:

- Assessed for eligibility (including the number eligible/ineligible*)
- Withdrawals*
- Lost to follow-up*
- Included in the analysis
- Excluded from the analysis*

*=including reasons

6.4 Baseline participant characteristics

The young woman baseline characteristics to be summarised include:

- Age (years)
- Site
- Gender identity
- Sex at birth
- Marital status
- Sexuality

- First language
- Ethnicity
- Highest educational attainment
- Day-to-day activities limited due to being a person with a disability.
- Presence of mental health problems
- Religion
- Accommodation
- Living situation

A draft young woman baseline characteristics table can be found in Appendix 2.

The mentor baseline characteristics to be summarised include:

- Age (years)
- Gender identity
- Sex at birth
- Marital status
- Sexuality
- First language
- Ethnicity
- Highest educational attainment
- Day-to-day activities limited due to being a person with a disability.
- Religion
- Main occupation
- Pre-existing personal and/or professional relationship with the young woman
- Living with the young woman they are mentoring

A draft mentor baseline characteristics table can be found in (Appendix 3).

7. Dataset

An extract of the full dataset will be provided to the trial statistician for the purposes of feasibility analysis and later for the purpose of outcome analysis. The co-lead investigators and the TM will help clean data and answer data queries during analysis.

8. Analysis

Outcomes are assessed at baseline, post-intervention (16 weeks; primary endpoint), and follow-up (12 months; secondary endpoint). Feasibility analysis will be performed on two outcome assessment points, baseline and the primary endpoint of post-intervention (16 weeks).

8.1 Analysis methods

Analyses will not be carried out until the trial database has been authorised as complete and final and all protocol deviations have been identified. Outcomes will be analysed collectively, except for the 12 month follow up data which will be analysed in the definitive trial, if the feasibility trial is found to be feasible.

The analyses will be performed by the trial statistician and the analysis output will be reviewed by the senior statistician. Box and whisker plots will be produced to explore the distribution of variables. Continuous normally distributed variables will be summarised using means, SDs, and the range (presented as the minimum and the maximum). Continuous non-normally distributed variables will be summarised using medians, interquartile ranges and ranges. For categorical variables, frequencies and percentages will be presented. The frequency and percentage of missing data will be tabulated. To address objective 1.1 (Table 4), frequencies and percentages for the following quantities will be calculated and compared against the pre-specified progression criteria (Table 5):

- Young women recruited during the pre-specified recruitment period.
- Young women identified during the recruitment process that are eligible and interested in participation.
- Young women allocated to HOPEFUL that complete 4 or more sessions.
- Young women that provide primary outcome data at the post-intervention assessment.

To address objective 2.1, the standard deviation of the Trait Hope Scale (primary outcome) in young women will be calculated.

Hypothesis tests of clinical outcomes to determine the effect of HOPEFUL will not be performed as this is not the focus of feasibility trials[2] (See Table 4 for the objectives). Similarly, hypothesis tests of baseline characteristics between arms will not be performed; if any differences are identified these will be reported. Stata 18SE or later will be used to conduct the statistical analysis.

8.2 Harms

There are no investigational medicinal products being used as part of TLFP. The reporting period for adverse events (AEs) covers the period from the point of randomisation until the final follow-up (12 months). AEs will be elicited in both arms at the 16-week and 12-month follow up assessments using a self-reported AEs Checklist that will be administered to participating NEET young women. In addition, potential adverse psychological effects arising directly from participation in the intervention arm will be elicited by an unblinded researcher in a telephone/video call using the modified Edinburgh Adverse Effects of Psychological Therapy Scale[11] (EDAPTS). The EDAPTS will be administered shortly after the 16-week and 12-month follow-up assessments. The TM or other unblinded researcher will additionally ask the mentors to complete the same scale shortly after the young woman's 16-week assessment, i.e., at the end of the intervention period, in their own 16-week questionnaire. Further, AEs may also be detected spontaneously via verbal report from young women participants or mentors during any contact with researchers.

AEs are defined as: any untoward medical or psychological occurrence, unintended disease or injury, or untoward clinical signs in a participant taking part in the trial, whether or not they are related to the intervention or trial procedures. A serious adverse event is an AE that is categorised as serious based on one (or more) of the following criteria:

- results in death
- is life-threatening

- requires inpatient hospitalisation or prolongation of existing hospitalisation
- results in persistent or significant disability/incapacity
- leads to foetal distress, foetal death, or consists of a congenital anomaly or birth defect
- necessitates medical or surgical intervention to prevent any of the above
- is otherwise deemed medically significant by the investigator.

Within TLFP, the following events will also be considered SAEs:

- suicide attempts, defined as any act of deliberate self-harm/injury where the participant had intent to end their life; suicide attempts are considered SAEs regardless of whether the resultant harm was life-threatening or required hospitalisation
- risk behaviours that require police involvement and/or arrest.
- crisis care involving the ambulance service and/or presentation to an accident and emergency (A&E) unit.

Notes:

- The term "life-threatening" in the definition of "serious" refers to an event in which the participant was at risk of death at the time of the event; it does not refer to an event which hypothetically might have caused death if it were more severe.
- Planned hospitalisations for a pre-existing condition, without a serious deterioration in health, would not be considered an AE or SAE.

Other ‘important medical events’ may also be considered serious if they jeopardise the participant or require an intervention to prevent one of the above consequences. The term "life-threatening" in the definition of "serious" refers to an event in which the participant was at risk of death at the time of the event; it does not refer to an event which hypothetically might have caused death if it were more severe.

An Adverse Reaction (AR) is an AE that is judged by either the reporting investigator or the sponsor as having a reasonable possibility of a causal relationship (e.g. definitely, probably, or possibly related) to the any aspect of the study procedure (e.g. research assessments) or the HOPEFUL intervention (delivered at any duration).

The degree of certainty on the relatedness of the study procedure or intervention and an adverse event will be classified as either “not related”, “unlikely”, “possible”, “probably” or “definitely”. AEs classified as possible, probable, and definitely related are considered an AR.

- Not related: No evidence of any causal relationship.
- Unlikely: There is little evidence to suggest there is a causal relationship (e.g., the event did not occur within a reasonable time). There is another reasonable explanation for the event (e.g., the patient’s clinical condition, other concomitant treatment).
- Possible: There is some evidence to suggest a causal relationship. However, the influence of other factors may have contributed to the event (e.g., the patient’s clinical condition, other concomitant treatments). Cases where relatedness cannot be assessed, or no information has been obtained, should be classified as possible.
- Probable: There is evidence to suggest a causal relationship and the influence of other factors is unlikely.
- Definitely: There is clear evidence to suggest a causal relationship and other possible contributing factors can be ruled out beyond reasonable doubt. (as defined above) that has resulted in any of the consequences characteristic of a Serious Adverse Event (as defined above). A Serious Adverse Reaction, the nature and severity of which is not consistent with the information known about the TLFP intervention or study procedures in the view of the investigator.

A Serious Adverse Reaction (SAR) is an AR that has resulted in any of the consequences characteristic of an SAE. A Suspected Unexpected Serious Adverse Reaction (SUSAR). A Suspected Unexpected Serious Adverse Reaction (SUSAR) is an SAR, the nature and

severity of which is not consistent with the information known about the TLFP intervention or study procedures in the view of the investigator.

Frequencies and percentages of each AE will be tabulated (Appendix 4). Visualisations proposed by Philips et al[12] may be used, as appropriate, to display safety data.

9. Appendix 1 The Trait Hope Scale

Within TLFP feasibility trial, the trait hope scale for a particular young woman would be considered complete if the 8-items that are involved in deriving the total hope scale score are completed (Table 7)[13].

Table 7: The Trait Hope Scale questionnaire items

Question no.	Question text	Agency item	Pathway item	Total Hope Scale score items*
1	I can think of many ways to get out of a jam.		✓	✓
2	I energetically pursue my goals.	✓		✓
3	I feel tired most of the time.			
4	There are lots of ways around any problem.		✓	✓
5	I am easily downed in an argument.			
6	I can think of many ways to get the things in life that are important to me.		✓	✓
7	I worry about my health.			
8	Even when others get discouraged, I know I can find a way to solve the problem.		✓	✓
9	My past experiences have prepared me well for my future.	✓		✓
10	I've been pretty successful in life.	✓		✓
11	I usually find myself worrying about something.			
12	I meet the goals that I set for myself.	✓		✓
Note: No.=Number. *When administering the scale, it is called The Future Scale. The agency subscale score is derived by summing items 2, 9, 10, and 12; the pathway subscale score is derived by adding items 1, 4, 6, and 8. The total Hope Scale score is derived by summing the four agency and the four pathway items.				

10. Appendix 2 Young woman baseline characteristics table

Note: The finalised form of the table may differ from the example given below.

Characteristic	HOPEFUL TOGETHER	HOPEFUL FUTURE	Total
Age (years) reported – Freq. (%)			
Median (IQR) [range]			
Local authority – Freq (%)			
Brighton & Hove			
East Sussex			
Kent			
Medway			
Norfolk			
Other			
Gender identity reported – Freq. (%)			
Woman/girl			
Transgender			
Non-binary			
I identify as another term			
Gender fluid*			
Prefer not to say			
Missing gender identity – Freq. (%)			
Biological sex assigned at birth– Freq. (%)			
Female			
Male			
Intersex			
Prefer not to say			
Missing biological sex assigned at birth – Freq. (%)			
Marital status– Freq. (%)			
Cohabiting			
In a long-term relationship			
Married/Civil Partnership			
Separated/divorced			
Single			
Widowed			
Prefer not to say			

Characteristic	HOPEFUL TOGETHER	HOPEFUL FUTURE	Total
Missing marital status– Freq. (%)			
Sexual orientation– Freq. (%)			
Bisexual			
Gay			
Heterosexual (Straight)			
I identify as another term			
Queer*			
Lesbian			
Prefer not to say			
Missing sexual orientation – Freq. (%)			
First language- Freq. (%)			
Missing first language - Freq. (%)			
Ethnicity- Freq. (%)			
Asian or Asian British (Indian, Pakistani, Bangladeshi, any other Asian background)			
Black or Black British (Caribbean, African, Any other Black background)			
Mixed (White and Black Caribbean, White and Black African, White and Asian, Any other mixed background)			
Other ethnic groups (Chinese, any other ethnic group)			
Roma*			
White (British, Irish, any other White background)			
Prefer not to say			
Missing ethnicity - Freq. (%)			
Highest educational attainment- Freq. (%)			
A-Level or equivalent			
GCSE or equivalent			

Characteristic	HOPEFUL TOGETHER	HOPEFUL FUTURE	Total
None			
Postgraduate or equivalent			
Undergraduate or equivalent			
Prefer not to say			
Missing education - Freq. (%)			
Day-to-day activities limited due to being a person with a disability - Freq. (%)			
No			
Yes - a little			
Yes - a lot			
Prefer not to say			
Missing day to day activities limited - Freq. (%)			
Religion- Freq. (%)			
Buddhist			
Christian (including Church of England, Catholic, Protestant and all other Christian denominations)			
Hindu			
Jewish			
Muslim			
Other			
Druid*			
Sikh			
Prefer not to say			
Missing religion - Freq. (%)			
Accommodation type - Freq. (%)			
Owner occupied			
Rented (private landlord)			
Rented (Local Authority/Housing Association)			
Supported accommodation (24 hour staffed)			

Characteristic	HOPEFUL TOGETHER	HOPEFUL FUTURE	Total
Supported accommodation (less than 24 hour staffed)			
Hospital			
Mobile accommodation, e.g., van or caravan			
Fixed/Static caravan			
Homeless/Sofa-surfing/Temporary Accommodation			
Other			
Hostel*			
Missing accommodation - Freq. (%)			
Living situation - Freq. (%)			
Live alone (with or without pets)			
Live with own children			
Live with others' children			
Live with partner(s)			
Live with own parent(s)			
Live with carer(s)			
Live with sibling(s)			
Live with other relative(s), e.g., aunt, uncle, grandparent, cousins			
Live with flatmates/housemates			
Live with friends			
Other			
Missing living situation - Freq. (%)			
Note: The entries beneath *I identify as another term* or *other category* are examples to show how the table may look. * = Described in the participant's own words. Freq. = Frequency.			

11. Appendix 3 Mentor baseline characteristics

Note: The finalised form of the table may differ from the example given below.

Characteristic	HOPEFUL TOGETHER	HOPEFUL FUTURE	Total
Age (years) reported – Freq. (%)			
Median (IQR) [range]			
Missing age – Freq. (%)			
Local authority of the young woman being mentored – Freq (%)			
Brighton & Hove			
East Sussex			
Kent			
Medway			
Norfolk			
Other			
Gender identity reported – Freq. (%)			
Man/boy			
Woman/girl			
Transgender			
Non-binary			
I identify as another term			
Gender fluid*			
Prefer not to say			
Missing gender identity – Freq. (%)			
Biological sex assigned at birth– Freq. (%)			
Female			
Male			
Intersex			
Prefer not to say			
Missing biological sex assigned at birth – Freq. (%)			
Marital status– Freq. (%)			
Cohabiting			
In a long-term relationship			

Characteristic	HOPEFUL TOGETHER	HOPEFUL FUTURE	Total
Married/Civil Partnership			
Separated/divorced			
Single			
Widowed			
Prefer not to say			
Missing marital status– Freq. (%)			
Sexual orientation– Freq. (%)			
Bisexual			
Gay			
Heterosexual (Straight)			
I identify as another term			
Queer*			
Lesbian			
Prefer not to say			
Missing sexual orientation – Freq. (%)			
First language- Freq. (%)			
Missing first language - Freq. (%)			
Ethnicity- Freq. (%)			
Asian or Asian British (Indian, Pakistani, Bangladeshi, any other Asian background)			
Black or Black British (Caribbean, African, Any other Black background)			
Mixed (White and Black Caribbean, White and Black African, White and Asian, Any other mixed background)			
Other ethnic groups (Chinese, any other ethnic group)			
Roma*			

Characteristic	HOPEFUL TOGETHER	HOPEFUL FUTURE	Total
White (British, Irish, any other White background)			
Prefer not to say			
Missing ethnicity - Freq. (%)			
Highest educational attainment- Freq. (%)			
A-Level or equivalent			
GCSE or equivalent			
None			
Postgraduate or equivalent			
Undergraduate or equivalent			
Prefer not to say			
Missing education - Freq. (%)			
Day-to-day activities limited due to being a person with a disability - Freq. (%)			
No			
Yes - a little			
Yes - a lot			
Prefer not to say			
Missing day to day activities limited - Freq. (%)			
Religion- Freq. (%)			
Buddhist			
Christian (including Church of England, Catholic, Protestant and all other Christian denominations)			
Hindu			
Jewish			
Muslim			
Other			
Druid*			
Sikh			
Prefer not to say			
Missing religion - Freq. (%)			
Main occupation - Freq. (%)			
Full-time employment			

Characteristic	HOPEFUL TOGETHER	HOPEFUL FUTURE	Total
Part-time employment			
Voluntary employment			
Full-time studying			
Part-time studying			
Homemaker			
Full-time parent or carer for children aged under 18 years			
Unwaged caring role for adults aged 18 years or over			
Unable to work or study due to being a person with a disability			
Looking for employment			
Other main occupation			
Prefer not to say			
Missing main occupation - Freq. (%)			
Type of employment- Freq. (%)			
Professional occupations, e.g., educational professionals, doctors, nurses and midwives			
Associate professional and technical occupations, e.g., artistic, literary and media occupations, welfare and housing professionals, marketing			

Characteristic	HOPEFUL TOGETHER	HOPEFUL FUTURE	Total
Administrative and secretarial occupations			
Skilled trade occupations			
Leisure and other service occupations			
Sales and customer service occupations			
Plant and machine operatives			
Elementary occupations, e.g., elementary cleaning and storage occupations			
Missing type of employment - Freq. (%)			
Pre-existing personal and/or professional relationship with the young woman- Freq. (%)			
Yes			
No			
Missing pre-existing personal and/or professional relationship with the young woman - Freq. (%)			
Pre-existing relationship with the young woman. - Freq. (%)			
Immediate family, e.g., parent, carer, sibling			
Wider family, e.g., aunt, uncle, cousin, grandparent			
Friend of the young woman			
Friend of the family			
Neighbour			
Teacher or teaching assistant			
Sports coach			

Characteristic	HOPEFUL TOGETHER	HOPEFUL FUTURE	Total
Community leader, e.g., scout leader, reverend			
Employment or education support worker			
Health or care worker, e.g., personal assistant, support worker, home carer			
Youth worker			
Employer			
Colleague			
Other			
Prefer not to say			
Missing pre-existing relationship with the young woman - - Freq. (%)			
Live with the young woman that you have been invited to mentor - Freq. (%)			
Currently living together			
Lived together in the past but not now			
Never lived together			
Prefer not to say			
Missing live with the young woman that you have been invited to mentor - Freq. (%)			
Note: The entries beneath *I identify as another term* or *other category* are examples to show how the table may look. * = Described in the participant's own words. Freq. = Frequency.			

12. Appendix 4 Adverse event and serious adverse event tables

Note: the finalised form of the table may differ from the examples given below.

	HOPEFUL TOGETHER						HOPEFUL FUTURE					Total					
Adverse event description	Events - Freq. (%)	Young woman - Freq. (%)	Mentor- Freq. (%)	Duration (days)	Out.*	Rel.†	Events - Freq. (%)	Young woman - Freq. (%)	Duration (days)	Out.*	Rel.†	Events - Freq. (%)	Young woman - Freq. (%)	Mentor- Freq. (%)	Duration (days)	Out.*	Rel.†

Note: *Out.= Outcome, †Rel. = Relatedness. Outcome is categorised as: Resolved, resolved with sequelae, ongoing, death and unknown. †Relatedness is categorised as: unrelated, unlikely, possibly and probably.

	HOPEFUL TOGETHER						HOPEFUL FUTURE					Total					
Serious adverse event description	Events - Freq. (%)	Young woman - Freq. (%)	Mentor- Freq. (%)	Duration (days)	Ser.§	Rel.†	Events - Freq. (%)	Young woman - Freq. (%)	Duration (days)	Ser.§	Rel.†	Events - Freq. (%)	Young woman - Freq. (%)	Mentor - Freq. (%)	Duration (days)	Ser.§	Rel.†

Note: §Ser. = Seriousness, †Rel. = Relatedness. Seriousness is categorised as: death, life threatening, Persistent or significant disability or incapacity, Injury/illness requiring immediate medical attention, Hospitalisation – Psychiatric A&E visit, Hospitalisation – Psychiatric involuntary admission or prolongation, Hospitalisation – Psychiatric voluntary admission or prolongation, Hospitalisation – Physical/general health A&E visit, Hospitalisation – Physical/general health admission or prolongation, Otherwise considered medically significant, Leads to foetal distress, foetal death or consists of a congenital anomaly or birth defect, Psychiatric crisis care (i.e., outpatient crisis team involvement), Risk behaviours that require police involvement and/or arrest, Any suicide attempt (with intent to end life), regardless of severity of injury. †Relatedness is categorised as: unrelated, unlikely, possibly and probably.

13. References

1. Gamble C, Krishan A, Stocken D, et al. Guidelines for the Content of Statistical Analysis Plans in Clinical Trials. *JAMA* 2017;318(23):2337-43. doi: 10.1001/jama.2017.18556
2. Eldridge SM, Chan CL, Campbell MJ, et al. CONSORT 2010 statement: extension to randomised pilot and feasibility trials. *BMJ* 2016;355:i5239. doi: 10.1136/bmj.i5239
3. Berry C, Hodgekins J, Michelson D, et al. A Systematic Review and Lived-Experience Panel Analysis of Hopefulness in Youth Depression Treatment. *Adolesc Res Rev* 2022;7(2):235-66. doi: 10.1007/s40894-021-00167-0 [published Online First: 20210706]
4. Murphy EC, Holmes C, Mayhew K. Not participating in education, employment or training (NEET): hope to mitigate new social risks in the UK? *Longit Life Course Stud* 2022;13(4):596-620. doi: 10.1332/175795921x16590816546869 [published Online First: 20220830]
5. Whitty C. Chief Medical Officer's Annual Report 2021 Health in Coastal Communities., 2021.
6. Berry C, Hodgekins J, French P, et al. Clinical and cost-effectiveness of social recovery therapy for the prevention and treatment of long-term social disability among young people with emerging severe mental illness (PRODIGY): randomised controlled trial. *Br J Psychiatry* 2022;220(3):154-62. doi: 10.1192/bjp.2021.206
7. Thornicroft G, Becker T, Knapp M, al e. CSRI European version. International Outcome Measures in Mental Health: Quality of Life, Needs, Service Satisfaction, Costs and Impact on Carers2006.
8. Snyder CR, Harris C, Anderson JR, et al. The will and the ways: Development and validation of an individual-differences measure of hope. *Journal of Personality and Social Psychology* 1991;60(4):570-85. doi: 10.1037/0022-3514.60.4.570
9. Teare MD, Dimairo M, Shephard N, et al. Sample size requirements to estimate key design parameters from external pilot randomised controlled trials: a simulation study. *Trials* 2014;15(1):264. doi: 10.1186/1745-6215-15-264
10. Arbon A, Dailey N, Perry N. Identifying and Reporting Deviations, Serious Breaches and Misconduct and

Use of File Notes Standard Operating Procedure. Brighton: Brighton and Sussex Clinical Trials Unit, 2022.
11. Mc Glanaghy E, Jackson JL, Morris P, et al. Discerning the adverse effects of psychological therapy: Consensus between experts by experience and therapists. *Clin Psychol Psychother* 2022;29(2):579-89. doi: 10.1002/cpp.2648 [published Online First: 20210721]
12. Phillips R, Cro S, Wheeler G, et al. Visualising harms in publications of randomised controlled trials: consensus and recommendations. *BMJ* 2022;377:e068983. doi: 10.1136/bmj-2021-068983

13. Snyder CR. Adult Hope Scale 2025 [Available from: https://ppc.sas.upenn.edu/resources/questionnaires-researchers/adult-hope-scale accessed 06/02/2025.
